# *PVT1* signals an androgen-dependent transcriptional repression program in prostate cancer cells and a set of the repressed genes predicts high-risk tumors

**DOI:** 10.1186/s12964-020-00691-x

**Published:** 2021-01-11

**Authors:** Alexandre Videira, Felipe C. Beckedorff, Lucas F. daSilva, Sergio Verjovski-Almeida

**Affiliations:** 1 Laboratório de Expressão Gênica Em Eucariotos, Instituto Butantan, Av. Vital Brasil 1500, São Paulo, SP 05503-900 Brazil; 2 Departamento de Bioquímica, Instituto de Química, Universidade de São Paulo, São Paulo, SP 05508-900 Brazil; 3 Present Address: Sylvester Comprehensive Cancer Center, University of Miami Miller School of Medicine, Miami, FL USA

**Keywords:** LincRNA *PVT1*, Prostate cancer, *PVT1* knockdown, Genome-wide transcriptional repression, Tumor suppressor genes

## Abstract

**Background:**

Androgen receptor (AR) and polycomb repressive complex 2 (PRC2) are known to co-occupy the loci of genes that are downregulated by androgen-stimulus. Long intergenic non-coding RNA (lincRNA) *PVT1* is an overexpressed oncogene that is associated with AR in LNCaP prostate cancer cells, and with PRC2 in HeLa and many other types of cancer cells. The possible involvement of *PVT1* in mediating androgen-induced gene expression downregulation in prostate cancer has not been explored.

**Methods:**

LNCaP cell line was used. Native RNA-binding-protein immunoprecipitation with anti-AR or anti-EZH2 was followed by RT-qPCR with primers for *PVT1*. Knockdown of *PVT1* with specific GapmeRs (or a control with scrambled GapmeR) was followed by differentially expressed genes (DEGs) determination with Agilent microarrays and with Significance Analysis of Microarrays statistical test. DEGs were tested as a tumor risk classifier with a machine learning Random Forest algorithm run with gene expression data from all TCGA-PRAD (prostate adenocarcinoma) tumors as input. ChIP-qPCR was performed for histone marks at the promoter of one DEG.

**Results:**

We show that *PVT1* knockdown in androgen-stimulated LNCaP cells caused statistically significant expression upregulation/downregulation of hundreds of genes. Interestingly, *PVT1* knockdown caused upregulation of 160 genes that were repressed by androgen, including a significantly enriched set of tumor suppressor genes, and among them *FAS*, *NOV/CCN3, BMF*, *HRK, IFIT2, AJUBA, DRAIC* and *TNFRSF21*. A 121-gene-set (out of the 160) was able to correctly predict the classification of all 293 intermediate- and high-risk TCGA-PRAD tumors, with a mean ROC area under the curve AUC = 0.89 ± 0.04, pointing to the relevance of these genes in cancer aggressiveness. Native RIP-qPCR in LNCaP showed that *PVT1* was associated with EZH2, a component of PRC2. *PVT1* knockdown followed by ChIP-qPCR showed significant epigenetic remodeling at the enhancer and promoter regions of tumor suppressor gene *NOV*, one of the androgen-repressed genes that were upregulated upon *PVT1* silencing.

**Conclusions:**

Overall, we provide first evidence that *PVT1* was involved in signaling a genome-wide androgen-dependent transcriptional repressive program of tumor suppressor protein-coding genes in prostate cancer cells. Identification of transcriptional inhibition of tumor suppressor genes by *PVT1* highlights the pathway to the investigation of mechanisms that lie behind the oncogenic role of *PVT1* in cancer.

**Video Abstract**

## Background

Prostate cancer (PCa) is the second most diagnosed type of cancer in men in the world [1]. Development of PCa is highly dependent on androgen receptor (AR) [2, 3], a transcription factor that induces expression activation or repression of prostate-specific genes through AR interaction with hundreds of coactivators or corepressors [4]. The ability of AR to repress transcription in LNCaP prostate cancer cells is related among other factors to cooperation with EZH2 histone-modifying enzyme, a component of the polycomb repressor complex 2 (PRC2) [5]. Specific AR-associated coregulators provide expression fine-tuning of hundreds of androgen-responsive target genes [6, 7], although the full complement of factors underlying expression activation or repression in the prostate have not yet been fully defined.

Long non-coding RNAs (lncRNAs) are pervasively transcribed in the human genome [8] and are recognized as an important layer of gene expression regulation [9]. Only a small number of lncRNAs have so far been functionally characterized, and their diverse roles include modification of chromatin states to increase or suppress transcriptional activation [10]. In 2004, we showed that in prostate cancer the expression of a set of antisense intronic non-coding RNAs correlates to the degree of tumor differentiation [11]. Over the past years, knowledge about lncRNAs involvement in prostate cancer has continuously progressed, and some three dozen lncRNAs have had their molecular mechanisms characterized in PCa [12, 13]. Understanding the complex network of gene regulation involving lncRNAs, and identifying their gene targets, will allow for their use in new strategies for diagnosis, prognosis and cancer therapy [14, 15].

Regarding the possible interplay between AR and lncRNAs, two studies have demonstrated AR association with *HOTAIR* [16], *PCGEM* and *PRCNR1* [17] lncRNAs in PCa. Our group has recently shown that in LNCaP cells the AR is significantly associated with over six hundred other lncRNAs [18]. Interestingly, the oncogenic lncRNA *PVT1* was one of the RNAs that was associated with AR, a finding that was not explored in that work [18]. *PVT1* is a long intergenic non-coding RNA (lincRNA) that is overexpressed in twenty-five different tumor tissues [19] including prostate adenocarcinoma [19, 20]. Clinical studies demonstrate that increased *PVT1* expression is correlated with shorter disease-free survival in prostate cancer [21] and with shorter overall survival in renal cell and colorectal carcinoma [22].

Knockdown of *PVT1* in prostate cancer cells and in other types of cancer cells resulted in decreased cell viability, induction of apoptosis and reduction in tumor volume [22], however the mechanisms of action of *PVT1* are largely unknown. *PVT1* has recently been shown in prostate cancer cells to act as a sponge for microRNA-186, thus increasing the expression of *Twist1* oncogene and promoting cell invasion and metastasis [23]. In fact, one of the mechanisms of *PVT1* function in prostate tumors, as well as in many other tumors, has been to act as a miRNA sponge in the cytoplasm [22]. Nevertheless, *PVT1* has also been described in prostate cancer to act in the nucleus to down-regulate miR-146a expression by inducing the methylation of CpG island in its promoter [24]. *PVT1* has been described in other types of cancer to act in the nucleus; thus, in ovarian cancer *PVT1* represses miR-214 expression by recruiting EZH2 to its promoter [25], and in non-small cell lung cancer (NSCLC) *PVT1* inhibits the expression of Large Tumor Suppressor Kinase 2 (*LATS2*) by recruiting EZH2 to the *LATS2* promoter [26]. In other tissues such as breast cancer [27], hepatocellular carcinoma [28], gastric cancer [29], and cholangiocarcinoma [30], *PVT1* has been shown to interact with EZH2 and epigenetically inhibit the expression of some target genes. In fact, no description of *PVT1* involvement with large-scale repression of gene expression has been published. *PVT1* has first been detected as one of the hundreds of lincRNAs physically associated in cervical cancer (HeLa cells) with the Polycomb repressive complex 2 (PRC2) proteins SUZ12 and EZH2 [31]. In that work, knockdown of six PRC2-associated lincRNAs, including *HOTAIR* and *TUG1*, has been shown to increase the expression of hundreds of genes known to be repressed by PRC2, suggesting that these lincRNAs function as expression silencers by regulating the epigenetic landscape in the cell [31]; however *PVT1* was not included as one of the six lincRNAs that were functionally characterized [31]. Here, we show that in LNCaP cells *PVT1* was associated with PRC2, besides being associated with AR. Also, using *PVT1* knockdown we show for the first time that in LNCaP cells *PVT1* was involved with the genome-wide regulation of gene expression. Interestingly, a set of androgen repressed genes increased their expression levels after *PVT1* knockdown, and tumor suppressor genes were enriched among them. We show that besides the known role of *PVT1* on regulating miRNA levels, this lincRNA acted to repress the transcription of hundreds of mRNAs in prostate cancer cells.

## Materials and methods

### Cell line and standard culture medium

Androgen-dependent LNCaP human prostate carcinoma cells (American Type Culture Collection, USA) were hormone-starved for 48 h in RPMI medium (Gibco, USA) supplemented with charcoal-stripped fetal bovine serum (CSS, Sigma-Aldrich, USA), and subsequently treated as described in the sections below. See Additional file 1: Methods for additional procedures and details.

### Native RNA-binding protein immunoprecipitation (RIP)

For native RIP [32] with LNCaP cells, CSS-supplemented medium was renewed, 10 nM synthetic androgen analog R1881 (Methyltrienolone, Sigma-Aldrich, USA) or vehicle (ethanol) were added, cells were incubated for an additional 24 h, and processed as described in the Magna RIP RNA-Binding Protein Immunoprecipitation Kit (Millipore, USA). See Additional file 1: Methods for additional procedures and details.

### Cell fractionation

For LNCaP cells fractionation, CSS-supplemented medium was renewed, 10 nM R1881 (or vehicle) was added, cells were incubated for an additional 24 h, and processed for subcellular fractionation by differential centrifugation [33]. See Additional file 1: Methods for additional procedures and details.

### *PVT1* knockdown

Androgen-starved cells were treated with lipofectamine 3000 complexed with a pool of PVT1_2 and PVT1_5 antisense LNA GapmeRs (150 pmol each, Exiqon-Qiagen, USA) designed against *PVT1* (Additional file 2: Table S1), or with a negative control scrambled GapmeR (300 pmol). For this, CSS-supplemented medium was renewed (5 mL medium), 30 μL lipofectamine 3000 complexed with 300 pmol LNA GapmeRs was added, and cells were incubated for 24 h. Subsequently, 1.0 nM R1881 or vehicle (ethanol) was added without changing the culture medium and cells were incubated for an additional 24 h. Cells were harvested with TRizol; total RNA was purified using RNeasy Mini Kit (Qiagen) according to the manufacturer's instructions.

### Dose-dependent effect of hormone on gene expression levels

CSS-supplemented medium was renewed and either 0.1, 1 and 10 nM R1881 or vehicle (ethanol) were added. After 24 h incubation, cells were harvested with TRizol; RNA was purified using RNeasy Mini Kit (Qiagen) according to the manufacturer’s instructions.

### Chromatin immunoprecipitation (ChIP)

*PVT1* was silenced by knockdown as described above, except that 900 pmol GapmeR was used (a pool of PVT1_2 and PVT1_5 (450 pmol each), or scrambled GapmeR at 900 pmol). After 24 h incubation, 10 nM R1881 was added, cells were incubated for additional 24 h, and processed with the Magna ChIP A/G kit (17-10085, Millipore) according to the manufacturer’s instructions. See Additional file 1: Methods for additional procedures and details.

### Reverse transcription and cDNA synthesis

In the RIP assay, 10 μL RNA sample from the immunoprecipitate were used for reverse transcription (in the expression assays, 1 μg total RNA was used instead) with SuperScript IV First Strand Synthesis System (Invitrogen, USA) and oligo-dT-(20) or random hexamer primers, according to the manufacturer's recommendations.

### Real-time quantitative PCR

qPCR was performed with specific primer pairs (Additional file 2: Table S1) and cDNA from the RIP or expression assays or DNA from the ChIP assay. See Additional file 1: Methods for additional procedures and details.

### Genome-wide gene expression analysis in LNCaP cells under *PVT1* silencing

Agilent SurePrint G3 Human Gene Expression v3 (8 × 60 k, G4851C) microarrays were used. Total RNA (200 ng) was obtained as described above in item *PVT1* knockdown, and RNA samples with a minimum RNA Integrity Number (RIN) > 8 were used. See Additional file 1: Methods for additional procedures and details.

### Statistical analyses

Significance Analysis of Microarrays (SAM) statistical test [34] was used with cutoff q-value ≤ 0.01 (i.e., a False Discovery Rate ≤ 1%) [35]. Genes with q-value ≤ 0.01 and |log2fold-change|> 1 were considered significantly differentially expressed. Gene Ontology analyses were performed with DAVID [36].

### Gene expression correlations in the TCGA dataset

The TCGA prostate adenocarcinoma (TCGA-PRAD) dataset was used and *PVT1*-related disease-free survival analysis was done with TANRIC tool [37]. To test the 121-gene-set as a tumor risk classifier, we implemented a machine learning Random Forest algorithm in python Scikit-Learn (v.0.20.2) [38] with gene expression data from all TCGA-PRAD tumors as input. See Additional file 1: Methods for additional procedures and details.

## Results

### *PVT1* associated with AR and with PRC2 in LNCaP cells

**Fig. 1:**
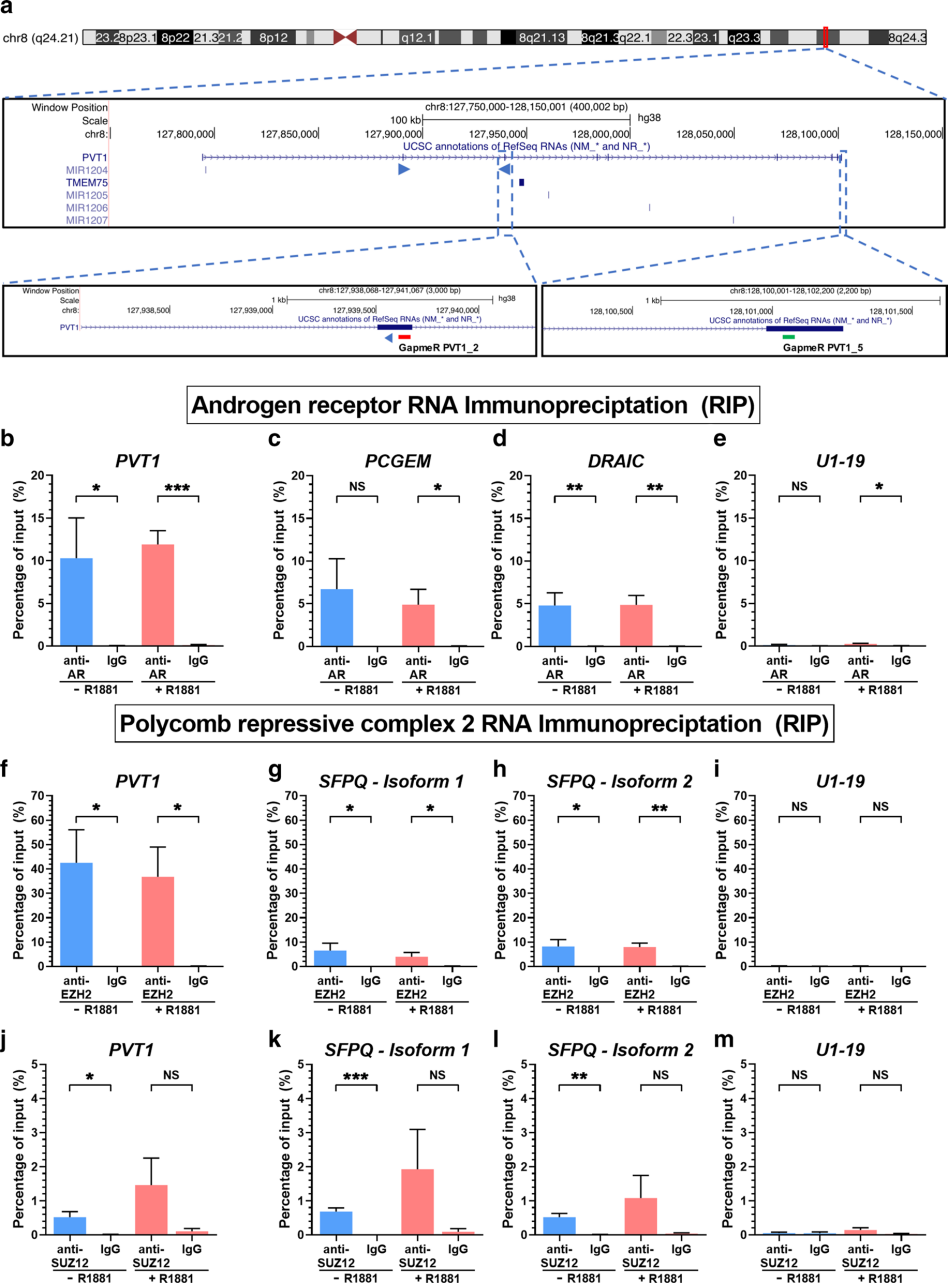
LincRNA *PVT1* associated both with AR and EZH2 in hormone-starved or in androgen-stimulated LNCaP cells. **a** Snapshot of the *PVT1* genomic locus on human Chromosome 8 showing the *PVT1* lincRNA, along with the pair of PCR primers (blue arrowheads) that was used for its quantification. The two lower insets show the locations of two antisense LNA GapmeR oligos (red and green blocks) that were used for *PVT1* knockdown in the experiments of Fig. 2. **b**–**e** RIP with anti-AR or nonspecific antibody (IgG), followed by RT-qPCR for genes *PVT1* (**b**, target), *PCGEM* (**c**, positive control), *DRAIC* (**d**), and *U1-19* (**e**, negative control). **f**–**i**, RIP with anti-EZH2 or nonspecific antibody (IgG), followed by RT-qPCR for *PVT1* (**f**, target), for two different isoforms of *SFPQ* (**g**, **h**, positive controls) and *U1-19* (**i**, negative control). **j**–**m** RIP with anti-SUZ12 or nonspecific antibody (IgG), followed by RT-qPCR for *PVT1* (**j**, target), for two different isoforms of *SFPQ* (**k**, **l**, positive controls) and *U1-19* (**m**, negative control). LNCaP cells under hormone-starved (-R1881, blue) or androgen-stimulated conditions (+ R1881, red) were tested. Data shown are mean ± s.e.m. of four biological replicates. T-test, *P < 0.05, **P < 0.01, ***P < 0.005

Human *PVT1* lincRNA (Accession NR_003367.3) is transcribed from chromosome 8 (q24.21) within a 400 kb-long locus (Fig. 1a), in a so-called “gene-desert” region where no protein-coding genes are transcribed [39].

We performed native RIP-qPCR in order to test if *PVT1* associated with AR and with PRC2 in LNCaP cells. *PVT1* was detected as significantly enriched in the anti-AR fraction relative to the non-specific IgG control in hormone-starved or androgen-stimulated cells (Fig. 1b, blue and red bars), similar to the positive control lncRNA *PCGEM* [17] (Fig. 1c, blue and red bars). Also interesting, prostate tumor suppressor lincRNA *DRAIC* [40], previously detected by us with RIP-seq as one of the other RNAs associated with AR [18], was confirmed here by RIP-qPCR (Fig. 1d, blue and red bars), whereas negative control snRNA *U1-19* was not associated with AR (Fig. 1e).

Next, we found *PVT1* as significantly enriched in the anti-EZH2 fraction relative to the non-specific IgG control, and again, the association with EZH2 occurred under hormone-starved and androgen-stimulated conditions (Fig. 1f, blue and red bars). Positive control lncRNA *SFPQ isoforms 1 and 2* [31] was significantly associated with EZH2 (Fig. 1g, h), whereas negative control snRNA *U1-19* was not (Fig. 1i).

RIP-qPCR was repeated against SUZ12, another component of PRC2, and *PVT1* showed a significant enrichment in the anti-SUZ12 fraction relative to the non-specific IgG control under hormone-starved conditions (Fig. 1j, blue bar). Under androgen stimulation (Fig. 1j, red bars), *PVT1* enrichment was non-significant. Similarly, positive control lncRNA *SFPQ isoforms 1 and 2* was significantly associated with SUZ12 only under hormone-starved conditions (Fig. 1k, l, blue bars), and not under androgen stimulation (Fig. 1k, l, red bars). Negative control snRNA *U1-19* was not associated with SUZ12 (Fig. 1m).

Association of *PVT1* with the two histone modifying enzymes raised the question about the subcellular localization of this lincRNA. We cultured LNCaP cells in the absence or presence of androgen, submitted them to subcellular fractionation, and we determined that *PVT1* was 98.5–98.8 localized in the nuclear fraction, and 1.2–1.5% in the cytoplasm (Additional file 2: Fig. S1).

### *PVT1* knockdown affected the expression of hundreds of genes in LNCaP cells

**Fig. 2:**
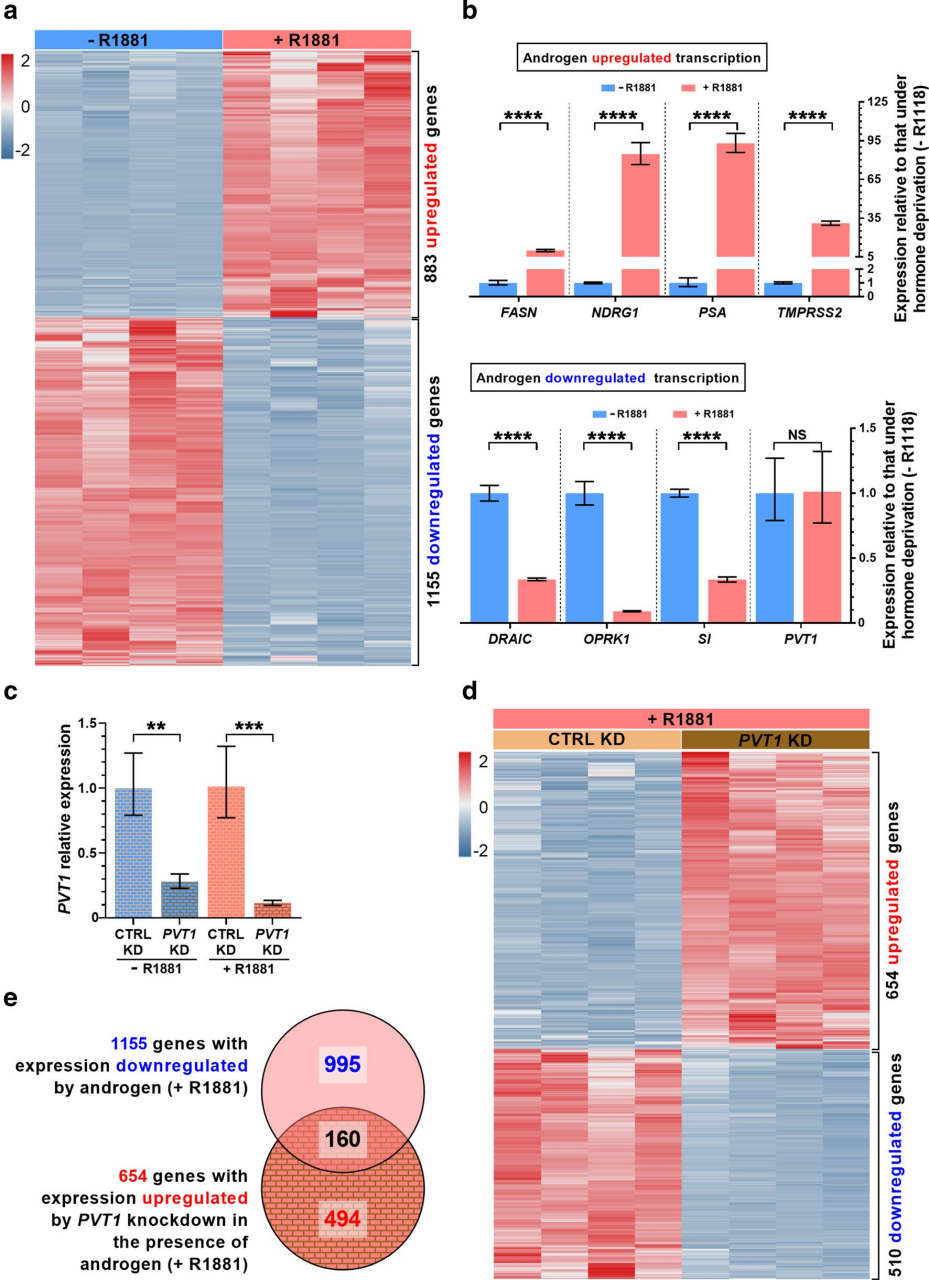
Androgen repressed the transcription of hundreds of genes and *PVT1* knockdown partially reversed this repression in LNCaP cells. LNCaP cells in culture were deprived of androgen (-R1881) or stimulated with 1 nM androgen (+ R1881), RNA was extracted from four biological replicates, purified and used. **a** Detection with microarrays of genome-wide statistically significant gene expression changes (one replicate in each column, one gene in each line; z-core color scale on the left) (q-value < 0.01, fold-change < 0.5 or fold-change > 2). **b** Validation with RT-qPCR of changes in expression of known androgen-responsive genes. **c** *PVT1* knockdown with a pool of two antisense LNA GapmeRs (*PVT1* KD) was efficient both in the presence (red) or in the absence (blue) of androgen, compared with a scrambled GapmeR (CTRL KD). **d** RNA was extracted from LNCaP cells after *PVT1* knockdown or CTRL knockdown and used for detection with microarrays of genome-wide statistically significant gene expression changes induced by *PVT1* knockdown (q-value < 0.01, fold-change < 0.5 or fold-change > 2). **e** Venn diagram shows that 160 genes were both downregulated by androgen and upregulated after *PVT1* knockdown under androgen stimulation. T-test, *P < 0.05, **P < 0.01, ****P < 0.001; mean ± s.e.m., four biological replicates

Given the finding that *PVT1* was associated with both AR and PRC2 in LNCaP, we verified if *PVT1* might be related to a genome-wide repression of gene expression in these cells under androgen stimulation. Initially, LNCaP cells were treated with androgen to establish the androgen-responsive genes. Expression-microarrays analyses identified 1155 genes significantly downregulated and 883 genes upregulated in androgen-stimulated compared with hormone-starved cells (Fig. 2a, Additional file 2: Table S2).

Control validation by RT-qPCR (Fig. 2b) showed that known androgen-induced genes *FASN*, *NDRG1*, *PSA* and *TMPRSS2* [5, 41] had their expression significantly upregulated by androgen (Fig. 2b, upper panel). Similarly, *DRAIC*, *OPRK1* and *SI*, known to have their expression repressed under androgen stimulation [5, 40, 41], were significantly downregulated by androgen (Fig. 2b, lower panel). Importantly, *PVT1* expression was not affected by androgen stimulation (Fig. 2b, lower panel).

Next, *PVT1* knockdown was induced in LNCaP cells with a pool of two antisense LNA GapmeRs, PVT1_2 (Fig. 1a, red block) and PVT1_5 (Fig. 1a, green block). This pool was able to effectively reduce *PVT1* expression to 30% or 10% of its endogenous level in hormone-starved (Fig. 2c, blue bars) or androgen-stimulated cells (Fig. 2c, red bars), respectively.

Finally, genome-wide expression-microarrays analyses showed that *PVT1* knockdown in androgen-stimulated LNCaP cells caused a significant upregulation of 654 genes and downregulation of 510 genes compared with their expression in control androgen-stimulated cells treated with a scrambled GapmeR (Fig. 2d, Additional file 2: Table S3).

A Gene Ontology (GO) analysis of the 654 upregulated genes showed a significant enrichment of “Regulation of RNA biosynthetic process” and “Regulation of gene expression” categories (Additional file 2: Fig. S2a). Noteworthy, GO categories related to tumor suppressor processes such as “Apoptotic process” and “Programmed cell death” were significantly enriched among the upregulated genes (Additional file 2: Fig. S2a), along with GO categories involved with “Negative regulation of cell proliferation” and “Negative regulation of developmental process” (Additional file 2: Fig. S2a).

Conversely, GO enrichment analysis of the 510 genes downregulated upon *PVT1* knockdown showed that “Protein kinase activity” along with “Regulation of GTPase activity” were the two most significantly enriched categories (Additional file 2: Fig. S2b), suggesting that *PVT1* in LNCaP cells might cause upregulation of genes involved in signaling pathways.

In hormone-starved cells, *PVT1* knockdown also caused a significant expression change of hundreds of genes (396 upregulated and 436 downregulated), compared with their expression in control hormone-starved cells treated with a scrambled GapmeR (Additional file 2: Fig. S3a, Table S4). “Cell proliferation” was one of the top most significantly enriched GO categories among the 396 upregulated genes (Additional file 2: Fig. S3b), also including “Positive regulation of cell cycle arrest”; this again suggests that endogenous *PVT1* in LNCaP cancer cells might repress the expression of genes related to cell cycle arrest and favor cell proliferation, even under hormone starvation. Among the 436 downregulated genes (Additional file 2: Fig. S3c), “Protein kinase” was one of the most significantly enriched GOs, again showing that endogenous amounts of *PVT1* in hormone-starved LNCaP cells might upregulate genes involved in signaling pathways.

Since *PVT1* associated with PRC2, we focused on the androgen-repressed genes and asked if *PVT1* knockdown would be able to increase the expression of a subset of those genes.

### *PVT1* knockdown restored the expression of a set of genes that were repressed by androgen in LNCaP cells

**Fig. 3:**
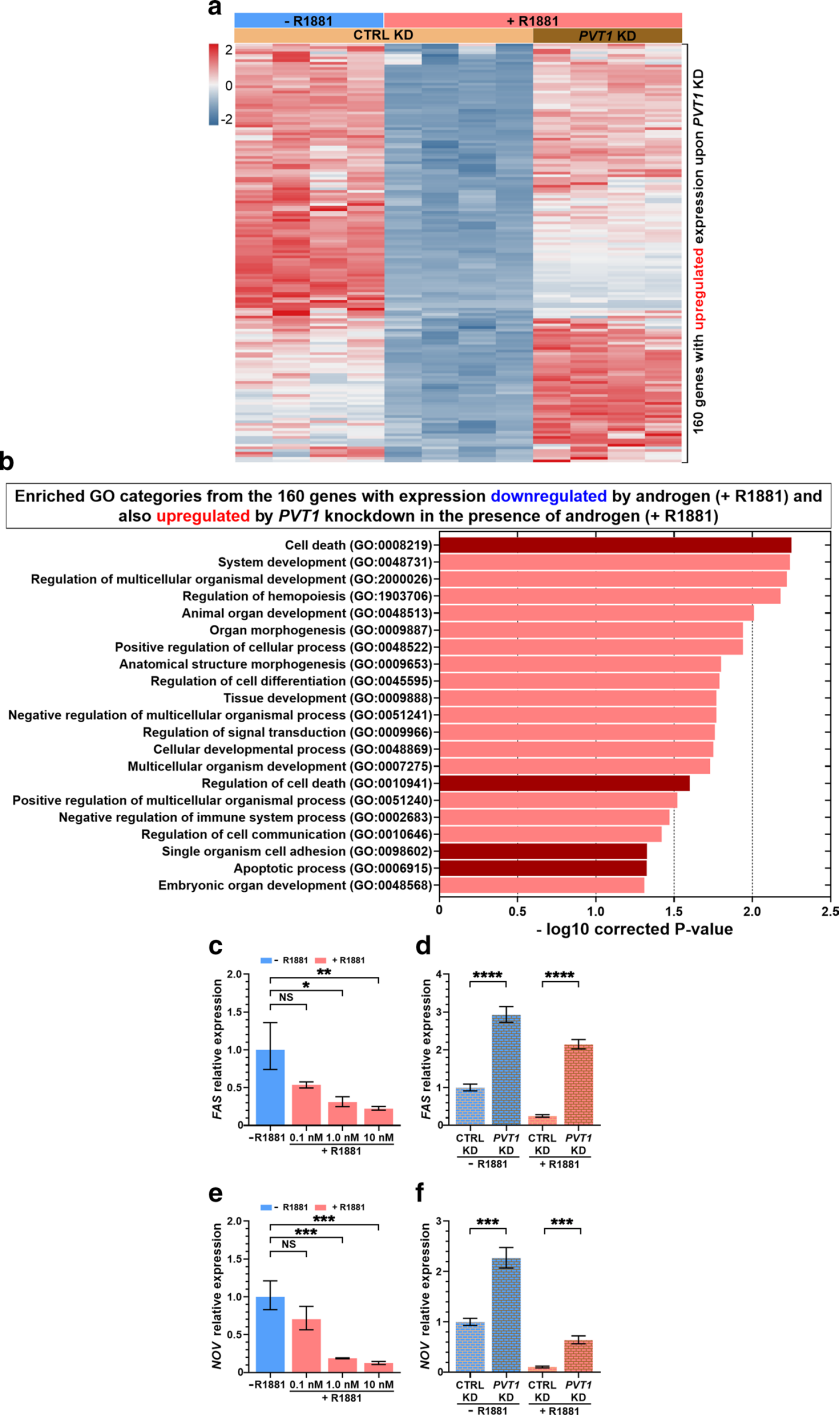
Genes de-repressed by *PVT1* knockdown in androgen-stimulated LNCaP cells were enriched in tumor suppressor functions. **a** Gene expression heatmap of the 160 genes (one in each line) that were significantly downregulated by androgen with endogenous *PVT1* (CTRL KD, + R1881) and were de-repressed (upregulated) by *PVT1* knockdown in androgen-stimulated cells (*PVT1* KD, + R1881) (q-value < 0.01, fold-change < 0.5 or fold-change > 2). For each condition indicated at the top, four biological replicates are shown (one in each column); z-score color scale is shown at left. **b** Gene Ontology terms significantly enriched (Benjamini–Hochberg corrected P < 0.05) with the 160 genes that were de-repressed by *PVT1* knockdown in androgen-stimulated cells. **c** Androgen dose-dependence of *FAS* gene expression. **d** Validation by RT-qPCR of the de-repression of *FAS* expression upon *PVT1* knockdown in androgen-starved (blue) or androgen-treated cells (red). **e** Androgen dose-dependence of *NOV* gene expression. **f** Validation by RT-qPCR of the de-repression of *NOV* expression upon *PVT1* knockdown in androgen-starved (blue) or androgen-treated cells (red). T-test, *P < 0.05, **P < 0.01, ***P < 0.005, ****P < 0.001; mean ± s.e.m., three biological replicates

To answer this question, we looked for overlap between androgen-responsive downregulated genes and genes whose expression was upregulated by *PVT1* knockdown under androgen stimulation. We identified 160 genes within the intersection between the two datasets (Fig. 2e, Additional file 2: Table S5). An expression heatmap shows that these 160 genes were all downregulated by androgen with endogenous levels of *PVT1* (Fig. 3a) and upon *PVT1* knockdown they were upregulated (Fig. 3a). It indicates that *PVT1* acted as a transcriptional repressor of these target genes, possibly involving AR and PRC2.

### Genes repressed by *PVT1* in LNCaP cells were related to tumor suppressor functions

GO enrichment analysis of the 160 genes whose expression was de-repressed by *PVT1* knockdown showed that a number of categories related to tumor suppressor functions were significantly enriched (Fig. 3b). The most significantly enriched GO was “Cell death”; also noteworthy were “Regulation of cell death”, “Single organism cell adhesion” and “Apoptotic process”.

Next, we validated by RT-qPCR the expression changes caused by *PVT1* knockdown in eight selected genes that are involved with either apoptosis or cell adhesion, out of the 160 genes repressed by androgen and de-repressed by *PVT1* knockdown. In parallel, to demonstrated that these eight genes could have a physiological relevance in the context of the androgen regulatory program, we stimulated LNCaP cells with different concentrations of synthetic androgen (0.1, 1 and 10 nM R1881) for 24 h (in the presence of endogenous levels of *PVT1*) and observed a dose-dependent target repression for all eight tested genes (Fig. 3c, e; Additional file 2: Fig. S4a to f).

*FAS* expression was repressed by androgen in the presence of endogenous *PVT1* (Fig. 3c) and after *PVT1* knockdown (Fig. 3d) its expression was restored both under hormone-starved (blue bars) and androgen-stimulated conditions (red bars). *FAS* tumor suppressor gene encodes a receptor protein that initiates the caspases cascade of cell death signals [42].

*NOV* expression was markedly repressed by androgen in LNCaP with endogenous *PVT1* (Fig. 3e). *PVT1* knockdown caused a two- to threefold increase in *NOV* expression (Fig. 3f), both in hormone-starved (blue bars) and in androgen-stimulated cells (red bars). *NOV* (nephroblastoma overexpressed, or *NOV/CCN3*) is a tumor suppressor gene encoding an extracellular-matrix protein that increases cell adhesion [43, 44].

The other six tumor suppressor genes tested by RT-qPCR were all de-repressed by *PVT1* knockdown (Additional file 2: Fig. S4a to f), namely *AJUBA* (or *JUB*), *BMF*, *DRAIC*, *IFIT2*, *TNFRSF21* and *HRK*, which indicates that *PVT1* acted as a mediator of transcriptional repression of a number of androgen-sensitive genes in LNCaP cells. Importantly, *TP53* tumor suppressor gene [45] was also found among the 654 genes that were upregulated upon *PVT1* knockdown in androgen-treated LNCaP (Additional file 2: Table S3), although *TP53* expression was not repressed by androgen.

Finally, in order to check if all of the above effects took place under unchanged *AR*, *EZH2* and *SUZ12* expression levels, i.e. to check if these genes were not themselves *PVT1* targets, we measured the mRNA levels of these genes in LNCaP cells after *PVT1* silencing (Additional file 2: Fig. S5a to d). Only *AR isoform 2* showed a significant 30% expression reduction after *PVT1* knockdown under androgen stimulation (Additional file 2: Fig. S5b), and the other genes tested here by RT-qPCR were not affected. Therefore, upregulation of the above 160 genes upon *PVT1* knockdown was not related to an increase in AR or to a decrease in PRC2 components EZH2 and SUZ12*.* Most likely, it was related to a change in occupancy of these epigenetic modifiers at target gene loci upon *PVT1* knockdown.

### The genes de-repressed by *PVT1* knockdown in LNCaP cells had their expression inversely correlated to patients’ prostate tumor risk classification

**Fig. 4:**
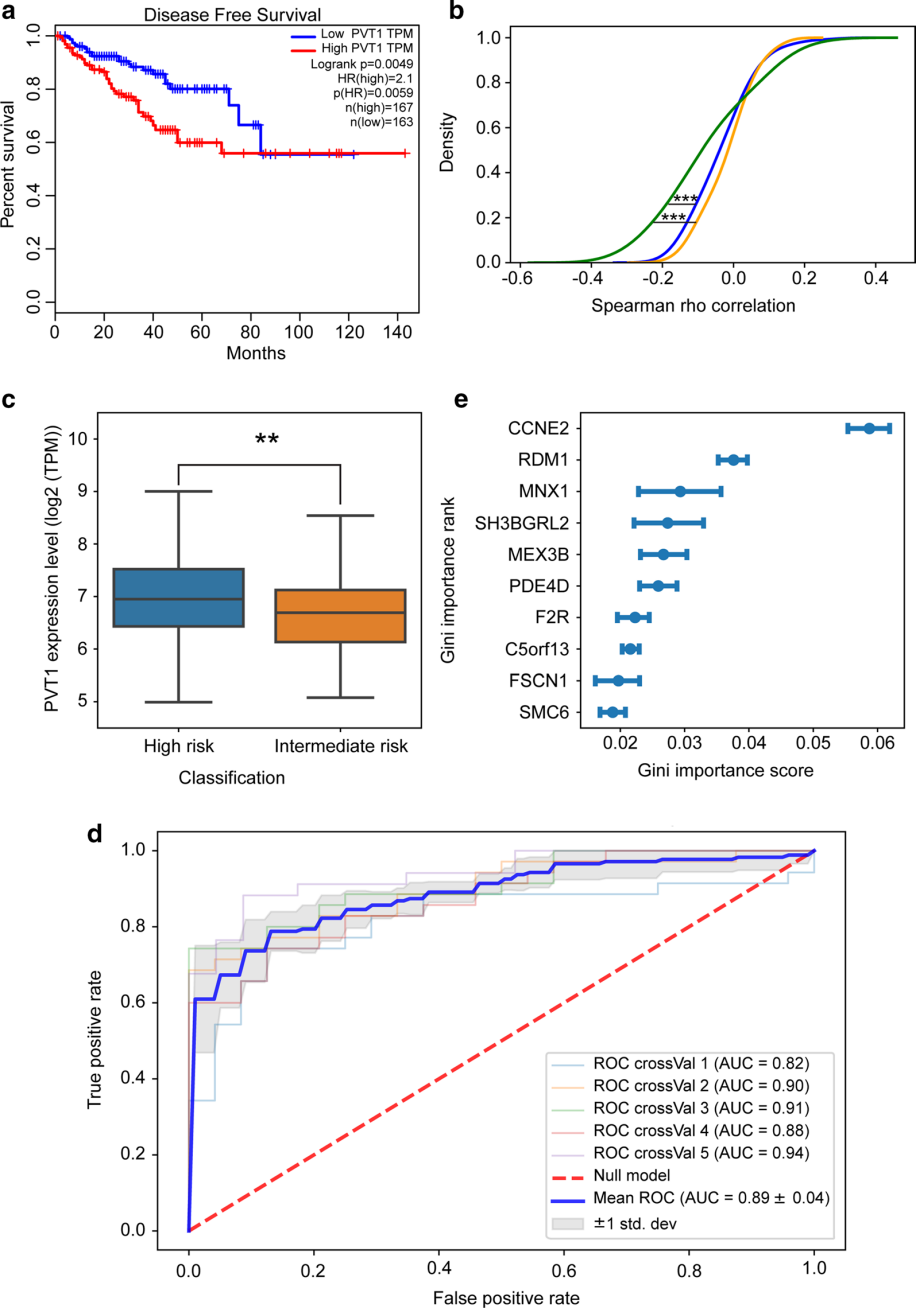
Genes de-repressed by *PVT1* knockdown in LNCaP cells had their expression in the TCGA prostate adenocarcinoma dataset correlated to tumor risk. **a** Kaplan–Meier disease-free survival analysis for TCGA-PRAD patients based on the *PVT1* expression level in the tumors of the first 3-quantile low-*PVT1* samples (blue, n = 163) and third 3-quantile high-*PVT1* samples (red, n = 167). **b** Cumulative density distribution of Spearman rho correlation between the expression levels of *PVT1* and each of the 121 genes upregulated after *PVT1* depletion in LNCaP cells. Correlation was calculated for each gene in the 121-gene-set across all TCGA-PRAD tumors from patients separated into three groups comprised of samples with low-, medium- or high-*PVT1* expression (orange, blue and green lines, respectively). The Kolmogorov–Smirnov test (two-tail ks-test) was applied to calculate the significance of the difference between the groups’ distribution (orange***, *P* value < 4.5 × 10^–6^; blue***, *P* value < 9.0 × 10^–4^). **c** *PVT1* expression level in TCGA-PRAD tumor samples with high (n = 174) and intermediate risk (n = 119). Significance was calculated using a two-tail t-test, **P < 0.01. **d** Classification of intermediate- and high-risk TCGA-PRAD tumors using a Random Forest machine-learning model. The ROC curves show the area under the curve (AUC) for the Random Forest model classification performance in a 5-way cross-validation (crossVal) scheme. Expression of the 121-gene-set across all intermediate- and high-risk TCGA-PRAD tumors were used as the input features for the machine-learning model. The 5-way cross-validation mean is indicated by the blue line, and the gray shade represents the validation standard deviation. **e** Rank of the top 10 most predictive genes in the Random Forest tumor classification analysis. Each blue point and error bars represent the mean ± S.D. of the Gini importance score for that gene, obtained in the 5-way cross-validation scheme

To evaluate the relevance of *PVT1* transcriptional repression in prostate cancer we analyzed RNA-seq data from 497 prostate adenocarcinoma samples (TCGA-PRAD). First, we determined that high *PVT1* levels were a statistically significant predictor of shorter disease-free survival when comparing patient tumors with high (33% highest) and low (33% lowest) *PVT1* expression levels (Fig. 4a).

Next, in the TCGA-PRAD dataset we identified 121 annotated genes (Additional file 2: Table S5) out of the 160 genes de-repressed by *PVT1* knockdown in LNCaP, and we retrieved their expression levels in all patient tumors. Patient tumors were clustered into three groups with low, medium and high levels of *PVT1* (33% quantiles), and the mean *PVT1* expression was significantly different among the groups (Additional file 2: Fig. S6a). We calculated the correlation between the expression of *PVT1* and the expression of each gene, across all patient tumors in each of the three groups. Expression of the 121-gene set was significantly more negatively correlated with *PVT1* in the high-*PVT1* tumors (Fig. 4b, green) compared with medium- and low-*PVT1* tumors (Kolmogorov–Smirnov test).

We identified in the TCGA-PRAD cohort the intermediate-risk tumors (n = 119; see Methods and Additional file 1: Methods) and the high-risk tumors (n = 174; see Methods and Additional file 1: Methods), and found that the expression of *PVT1* in the high-risk group was significantly (t-test p = 0.01) higher than in the intermediate-risk group (Fig. 4c).

In order to test the 121-gene-set as a tumor risk classifier, we trained a machine learning algorithm using the 121-genes expression data from 80% of all 293 intermediate- and high-risk TCGA-PRAD tumor samples. Next, we classified the tumors of the remaining 20% patients in this independent validation group, repeating the procedure in a 5-way cross-validation test, each with different patients in the 20% validation group (and therefore, each with a different training set corresponding to the 80% remaining samples). The 121-gene-set was able to correctly predict all 293 intermediate- and high-risk TCGA-PRAD tumors, with a mean ROC area under the curve AUC = 0.89 ± 0.04 (0.82–0.94) (Fig. 4d). The top 10 most important classifier genes (highest Gini scores) are shown in Fig. 4e; importance of all genes for the machine-learning classification is in Additional file 2: Fig. S6b.

### Knockdown of *PVT1* caused an epigenetic remodeling in the *NOV* gene enhancer and promoter regions

**Fig. 5:**
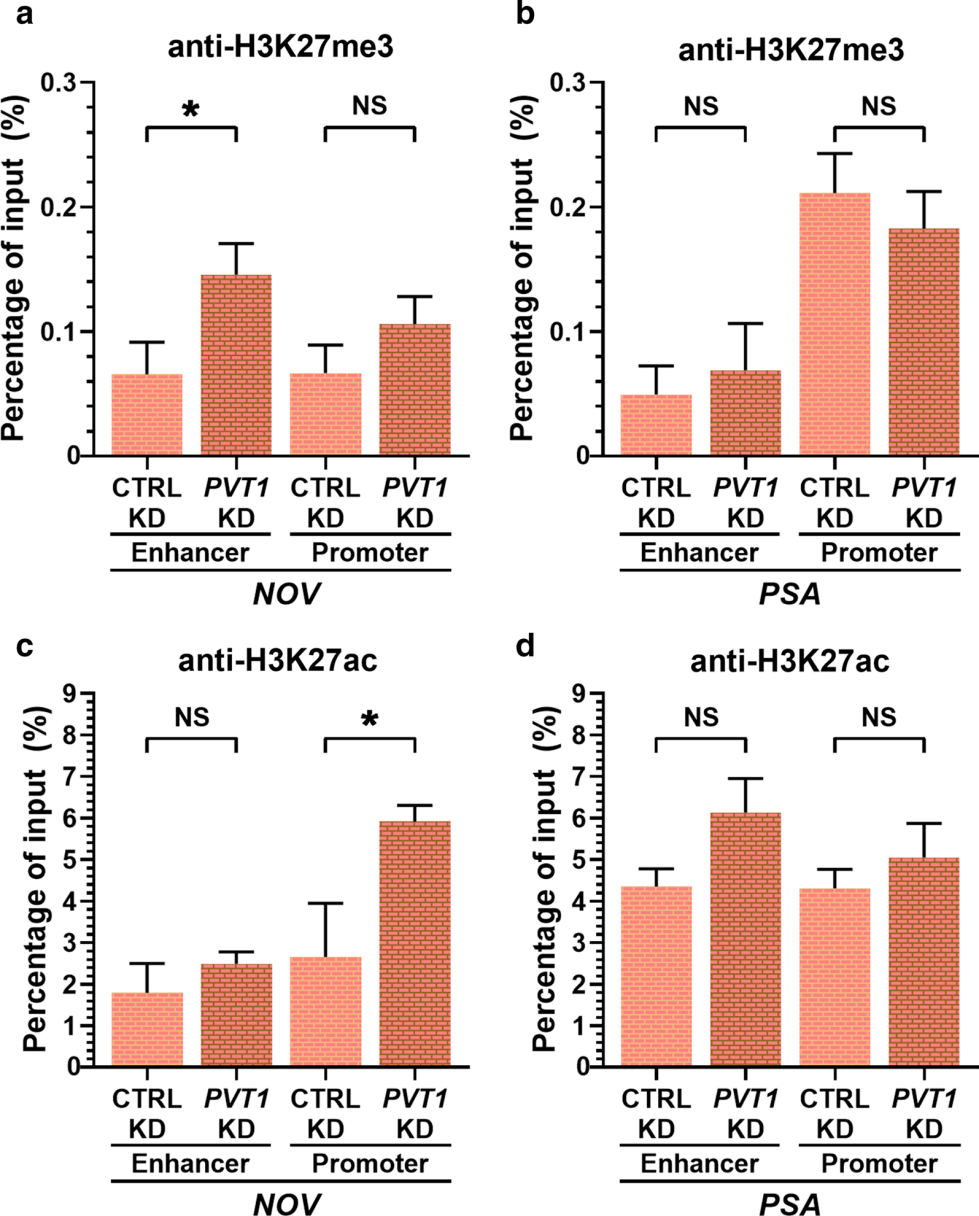
Epigenetic marks at the enhancer and promoter genomic regions of the *NOV* gene were remodeled upon *PVT1* knockdown. LNCaP cells in culture with androgen were treated with a pool of two antisense LNA GapmeRs targeting *PVT1* (*PVT1* KD) or with a scrambled GapmeR (CTRL KD), cells were then lysed and submitted to the Chromatin Immunoprecipitation (ChIP) protocol. **a**, **b** ChIP with anti-H3K27me3 Ab; or **c**, **d** with anti-H3K27ac Ab. The co-precipitated DNA was subjected to qPCR with primers for the enhancer (left two bars) and promoter (right two bars) genomic regions of the gene indicated at the bottom of each panel, either the *NOV* or the *PSA* gene. T-test, *P < 0.05, mean ± s.e.m. of three biological replicates

We have chosen the *NOV* gene locus for mapping the possible changes in epigenetic marks occurring upon *PVT1* knockdown because *NOV* is one of the most strongly repressed genes by AR and EZH2 in LNCaP cells [46] and upon AR occupancy of the enhancer at 63-kb upstream of the transcriptional start site (TSS) of *NOV*, a DNA loop occurs, bringing the enhancer closer to the promoter region of the *NOV* gene [46]. Then, AR recruits the EZH2 that catalyzes the trimethylation of histone H3 in lysine 27 around the *NOV* promoter, leading to inhibition of expression via epigenetic silencing [46]. Our hypothesis was that if *PVT1* lincRNA interacted with AR and EZH2, knockdown of *PVT1* would change the epigenetic landscape in the *NOV* gene locus. In parallel, as a non-related gene whose expression was not affected by *PVT1* knockdown, we looked at the epigenetic marks in the *PSA* gene locus. After *PVT1* knockdown there was a significant increase in H3K27me3 occupancy at the *NOV* enhancer (Fig. 5a) and a significant increase in H3K27ac occupancy at the *NOV* promoter (Fig. 5c). No significant change in occupancy of these histone marks was observed in the negative control gene *PSA* (Fig. 5b, d). The histone mark H3K27ac is associated with increased activation of gene transcription, primarily localizing around the TSS of human genes [47]. The increase in H3K27ac mark occupancy is in line with the activation of *NOV* transcription when *PVT1* was silenced (Fig. 3f).

## Discussion

This report is the first direct investigation of the genome-wide gene expression regulation effected by *PVT1* in prostate cancer cells. First, we identified that *PVT1* was associated in LNCaP cells both with AR and EZH2, a component of the polycomb complex, suggesting that *PVT1* could be potentially important for the androgen-induced repression program. In fact, *PVT1* could act as a scaffold for protein complexes, possibly acting in a similar way as *HOTAIR* lincRNA [48], which is important for driving two repressive complexes, histone methyltransferase PRC2 and histone demethylase LSD1 to their specific targets [48]. Next, we showed that *PVT1* knockdown in LNCaP cells affected the expression of over one thousand genes, indicating that a complex change in the gene expression program occurred upon *PVT1* silencing. In fact, the majority of the affected genes (56%) were upregulated upon *PVT1* silencing, indicating that *PVT1* predominantly acted as a transcriptional repressor. *PVT1* could possibly be one of the mediators of the previously reported role of AR as a global transcriptional repressor in LNCaP cells [5], where AR and EZH2 co-occupy the regulatory and promoter regions of androgen-repressed genes [5].

The smaller set of downregulated genes upon *PVT1* silencing (44% of the affected genes) may have their expression modulated through the *PVT1*-miRNA-mRNA axis, whereby the well documented miRNAs sponging role of *PVT1* would control the levels of critical miRNAs that target specific mRNAs in the cell [22]. The novel genome-wide transcriptional repressive role of *PVT1* lincRNA on protein-coding genes, which was uncovered by our experimental results, points to an additional mechanism of action whose future detailed characterization is warranted.

Most interestingly, among the genes upregulated by *PVT1* knockdown, a number of enriched GO categories were related to apoptosis and programmed cell death, either looking at the entire set of 654 upregulated genes or at the subset of 160 genes that had been repressed by androgen-stimulation and were de-repressed by *PVT1* silencing. “Cell adhesion” was another relevant enriched GO, among the genes upregulated by *PVT1* silencing. This indicates that the endogenous level of *PVT1* in LNCaP cells resulted in the repression of tumor suppressor genes involved with apoptosis and cell adhesion, which is in line with the known oncogenic role of *PVT1* [19]. Overall, these results suggest that increased levels of endogenous *PVT1*, known to occur in prostate cancer [19, 20]*,* could lead to downregulation of tumor suppressor genes and favor cell proliferation.

Knockdown of *PVT1* in prostate cancer cell lines has been shown to increase the abundance of activated cleaved caspase-9 and -3 proteins and to induce cell apoptosis [21]. Here, we identified with microarrays and confirmed by RT-qPCR that *PVT1* knockdown increased the expression of *FAS*, which is the pro-apoptotic gene encoding the membrane receptor that triggers the caspases activation cascade [42]. Low *FAS* expression has been described in LNCaP and PC3 cells [49, 50], however correlation between *FAS* and *PVT1* expression was not known until now.

We also showed here that *PVT1* knockdown did upregulate the expression of *NOV/CCN3*, the tumor suppressor gene encoding a protein that induces apoptosis in fibroblasts [51]. The *NOV/CCN3* gene regulates cell adhesion, migration, proliferation, differentiation and survival [52, 53]. *NOV/CCN3* decreased expression was already described in various tumor types and cancer cell lines including prostate cancer LNCaP cells [43, 44, 46, 54]. We observed that after *PVT1* knockdown there was a significant increase in H3K27me3 occupancy at the *NOV/CCN3* enhancer and a significant increase in H3K27ac occupancy at the *NOV/CCN3* promoter. Using *NOV/CCN3* as a model gene, Wu et al. [46] demonstrated that in LNCaP and VCaP cells, AR directly binds the enhancers of target genes and controls the promoter through DNA looping with enhancer. In addition, they showed that, while the enhancer elements are important in recruiting AR, the promoter dictates gene repression through repressive chromatin remodeling that is mediated by the histone H3 lysine 27 (H3K27) methyl-transferase EZH2 [46]. The increase in activating H3K27ac mark occupancy at the *NOV/CCN3* promoter that we observed here upon *PVT1* reduction, in parallel with an increased H3K27me3 occupancy at the *NOV/CCN3* enhancer, suggest that the lincRNA could drive the DNA looping, and that in its absence the promoter became free from the EZH2 deposition of methylation marks at the promoter region. In this context, it is interesting to note that upon *PVT1* decrease, the *NOV/CCN3* locus with increased H3K27me3 at the enhancer has assumed a poised enhancer configuration [55, 56]. It is tempting to speculate that a wider epigenetic reprograming has taken place, which could as well include DNA methylation that is known to reversibly regulate H3K27ac and H3K27me3 at tissue-specific enhancers [57]. Identification of additional genome-wide changes in epigenetic marks induced by *PVT1* knockdown will be an interesting line for future investigation.

While NOV/CCN3 is known as a matricellular, secreted protein, a cytoplasmic form of the NOV/CCN3 protein has been shown in prostate cancer LNCaP cells to interact with the N-terminal domain of the AR protein to inhibit nuclear translocation of AR, thereby acting in a negative feedback loop to block AR function [58]. In LNCaP cells, NOV/CCN3 physically interacts with the AR protein to retain it in the cytoplasm, thus reducing AR nuclear translocation, and *NOV/CCN3* overexpression strongly suppresses LNCaP growth program [58]. Of clinical relevance, *NOV/CCN3* depletion is a main driver of AR signaling and androgen-independent Castration Resistant Prostate Cancer (CRPC) progression and drug resistance; a significant decrease of *NOV/CCN3* expression is observed in CRPC compared with localized prostate cancer in various prostate cancer patient cohorts [58]. We suggest that increased levels of *PVT1* in advanced prostate cancer tumors could modulate an enhanced epigenetic inhibitory effect of AR on *NOV/CCN3* expression, through the AR-EZH2-*PVT1* axis.

Other pro-apoptotic genes that were found upregulated upon *PVT1* knockdown were *BMF*, which encodes a pro-apoptotic BCL2-family protein [59], *IFIT2*, which triggers apoptosis through a BCL2-dependent mitochondrial pathway [60], *TNFRSF21*, an apoptosis activator gene in bladder cancer [61], and *HRK*, which encodes a pro-apoptotic protein that interacts with BCL2 and Bcl-XL apoptosis-repressor proteins [62]. *PVT1* knockdown also upregulated the expression of *AJUBA* (or *JUB*), which encodes a cell–cell junction protein [63] whose low expression is observed in metastatic prostate cancer [64], of *DRAIC* lncRNA, a tumor suppressor gene that represses migration and cell invasion in LNCaP cells [40], and of tumor suppressor *TP53*, a master regulator of stress-induced FAS-dependent apoptosis [65]. Overall, our data indicate that in LNCaP cells *PVT1* caused transcriptional repression of an important set of tumor suppressor genes, and suggest that the increased expression of *PVT1* in advanced prostate cancer types [19, 20] may contribute to the aggressive phenotype by reducing apoptosis and cell–cell adhesion.

Our data show correlation of high-*PVT1*-expression tumors with shorter disease-free patient survival in the 497-patients TCGA-PRAD cohort, confirming the results of a previous publication [21] with a 152-Chinese-patients cohort. Also, we detected an inverse correlation in the TCGA-PRAD tumors between the expression of *PVT1* and the expression of the 121-gene-set (out of the 160 genes that were repressed by androgen and de-repressed by *PVT1* knockdown in LNCaP cells), which points to the relevance of further characterizing the interaction between *PVT1* and tumor suppressor genes in determining an aggressive phenotype in prostate cancer. In fact, the ability of the 121-gene-set expression profile to correctly predict tumor risk in the 293-patients intermediate- and high-risk TCGA-PRAD cohort (AUC = 0.89 ± 0.04), indicates that the role of this set of genes in prostate cancer progression should be the subject of further investigation.

## Conclusions

In prostate cancer cells, we show a novel genome-wide androgen-related role of *PVT1* lincRNA in signaling the transcriptional repression of protein-coding genes, and we show that the *PVT1*-targeted repressed gene set was enriched in tumor suppressor functions. This may lie behind the known aggressive phenotype of tumors expressing high levels of *PVT1* oncogene, which highlights the pathway towards further investigation of the mechanisms that link *PVT1* and its tumor suppressor targeted genes. We also show that the expression profile of a repressed 121-gene-set can be used with high confidence as a predictor of prostate-cancer patients’ high-risk tumors.

## Supplementary Information

**Additional file 1:** Supplementary Methods.

**Additional file 2:** Supplementary Figures and Tables.

## Data Availability

The microarray gene expression datasets generated for this study can be found in the GEO DataSets (https://www.ncbi.nlm.nih.gov/gds/) under accession number GSE133372.

## Abbreviations

AR: Androgen receptor
AUC: Area under the curve
ChIP-qPCR: Chromatin immunoprecipitation followed by quantitative PCR
crossVal: Cross-validation test
CSS: Charcoal-stripped fetal bovine serum
DEG: Differentially expressed gene
FDR: False Discovery Rate
GapmeR: 16-Nucleotides-long oligonucleotide enriched with LNA in the flanking regions and DNA in an LNA-free central gap
GO: Gene Ontology
KD: Knockdown
lincRNA: Long intergenic non-coding RNA
LNA: Locked nucleic acid
lncRNA: Long non-coding RNA
PCa: Prostate cancer
PRC2: Polycomb repressive complex 2
qPCR: Quantitative PCR
RIN: RNA Integrity Number
RIP-qPCR: RNA-binding-protein immunoprecipitation followed by quantitative PCR
ROC: Receiver operating characteristic
RT-qPCR: Reverse transcription followed by quantitative PCR
SAM: Significance Analysis of Microarrays
TCGA-PRAD: The Cancer Genome Atlas-Prostate adenocarcinoma
