# Supplementary material for: PVT1 signals an androgen-dependent transcriptional repression program in prostate cancer cells and a set of the repressed genes predicts high-risk tumors
Source: Cell Commun Signal. 2021 Jan 11;19:5. doi: 10.1186/s12964-020-00691-x (PMC7798249; doi:10.1186/s12964-020-00691-x)
Supplement: Supplementary file 2 — Additional file 1: Supplementary Methods. [file 12964_2020_691_MOESM2_ESM.pdf]

Supplementary Methods

Cell line and standard culture medium

Androgen-dependent LNCaP human prostate carcinoma cells (from the American Type Culture Collection) were cultured at 37 °C, 5 % CO_2 in RPMI medium (Gibco) supplemented with 10 % fetal bovine serum (Vitrocell), 4.5 g/L glucose, 1.5 g/L sodium hydrogen carbonate, 2.4 g/L HEPES, 1 % sodium pyruvate and penicillin (100 U/ml)/streptomycin (100 U/ml). Cells were grown until reaching approximately 50 % confluence, the standard medium was replaced by RPMI supplemented with charcoal-stripped fetal bovine serum (CSS, Sigma-Aldrich), which was changed twice, once every 24 h, totaling 48 h of hormone starvation. Subsequently, hormone-starved cells were subjected to different treatments as described under Materials and Methods in the main text.

Native RNA-binding protein immunoprecipitation (RIP) followed by RT-qPCR

For native RIP with LNCaP cells [1], CSS-supplemented medium was renewed, 10 nM synthetic androgen analog R1881 (Methyltrienolone, Sigma-Aldrich) or vehicle (ethanol) were added, cells were incubated for additional 24 h, and processed as described in the Magna RIP RNA-Binding Protein Immunoprecipitation Kit (Millipore). After those 24 h, the number of cells was approximately 2 x 10^7 for each antibody assay in each biological replicate, as estimated by Neubauer chamber cell counting. Cells were washed twice with ice-cold PBS, collected by cell scraper and centrifuged at 1500 rpm for 5 min at 4 °C. Cells pellets were resuspended in RIP lysis buffer as described in the Magna RIP RNA-Binding Protein Immunoprecipitation Kit (Millipore). Subsequent RIP steps were performed according to the manufacturer's instructions. Merck-Millipore antibodies used were anti-SUZ12 (03-179), anti-EZH2 (17-662), non-immunized mouse IgG (12-371), anti-AR (06-680) and non-immunized rabbit IgG (PP64B). Co-immunoprecipitated RNAs and in parallel the RNAs in the input samples were extracted with TRIzol (Invitrogen) followed by purification with RNeasy Micro kit (Qiagen) according to the manufacturer's instructions; RNA was eluted in 25 µl DEPC water. The use of Human Gene *RNVU1-19* – variant U1 small nuclear 19 (snRNA *U1-19*), RefSeq NR_104086 [2] as negative control was based on the Magna RIP RNA-Binding Protein Immunoprecipitation Kit (Millipore, USA) protocol. Using the Primer3 online tool [3] we have designed primer pairs specific for *RNVU1-18* and *RNVU1-19*, and we measured their expression levels in LNCaP along with the expression level of snRNA *U1* obtained with the primers from the Magna RIP kit. We chose *RNVU1-19*, which had in the input fraction a Ct value range (18.5 – 20.0) that was in the same range of values of *GAPDH* (17.2 – 19.0) and *ACTB* (18.0 – 19.3), and lower than the Ct values of *PVT1* (26.5 – 28.5) in the input.

Cell fractionation

For LNCaP cells fractionation, CSS-supplemented medium was renewed, 10 nM R1881(or vehicle) was added, cells were incubated for an additional 24 h, and processed for subcellular fractionation by differential centrifugation [4]. After nuclear and cytosolic fractions separation, RNA from each fraction was extracted with TRIzol (Invitrogen) followed by purification with RNeasy Micro kit (Qiagen) according to the manufacturer's instructions. RNA expression levels were measured by Real-time RT-qPCR as described below. *GAPDH* was used as a cytosolic marker, and *snRNA U1-19* was used as a nuclear marker, according to Yu et al. 2018 [5]. Three biological replicates of each condition, with three technical replicates of qPCR per sample were assayed.

Chromatin immunoprecipitation (ChIP) followed by qPCR

PVT1 was silenced by knockdown as described above, except that 900 pmol GapmeR was used (a pool of PVT1_2 and PVT1_5 (450 pmol each), or scrambled oligo at 900 pmol). After 24 h incubation, 10 nM R1881 was added, cells were incubated for additional 24 h, and processed with the Magna ChIP A/G kit (17-10085, Millipore) according to the manufacturer's instructions. Thus, culture medium was replaced with 20 mL crosslinking buffer (50 mM Hepes-KOH, pH 7.5, 100 mM NaCl, 1 mM EDTA, 0.5 mM EGTA) and crosslinking was carried with 1% formaldehyde for 10 min at room temperature. The reaction was stopped by incubation for 5 min with 125 mM glycine. Cells were washed twice with ice-cold PBS, once with ice-cold PBS supplemented with protease inhibitors and the cell pellet was rapidly frozen in liquid nitrogen. Cells were thawed on ice and processed with the Magna ChIP A/G kit (17-10085, Millipore) according to the manufacturer's instructions. Chromatin was fragmented using the Covaris S2 sonicator in a 130 µL tube under the following conditions: duty cycle 2 %, intensity 3, cycles per burst 200, time 480 s. ChIP was performed with 5 µg of each of the antibodies from Millipore: anti-H3K27ac (07-360), anti-H3K27me3 (07-449). Three biological replicates were assayed for each antibody.

Real-time quantitative PCR

qPCR was performed with primers specific for each gene as described in Supplementary Table S1 and cDNA from the RIP or expression assays (diluted 1:5 to 1:7) or DNA from the ChIP assay. The LightCycler 480 II equipment (Roche) and SYBR Green I Master Mix (Roche) were used. Three biological replicates were assayed in three technical replicates each. The delta-Ct (ΔCt) method was used, and the geometric mean Ct of constitutive genes *GAPDH* and *ACTB* was used as normalizer in gene expression assays. In RIP and ChIP assays, the amount of RNA or DNA in the input sample (before immunoprecipitation, measured in an aliquot corresponding to 10 % and 1 % of the volume, respectively) was used as normalizer.

Genome-wide gene expression analysis in LNCaP cells under *PVT1* silencing

Agilent SurePrint G3 Human Gene Expression v3 (8 x 60k, G4851C) microarrays were used. Total RNA (200 ng) was obtained as described in the main text in item *PVT1* knockdown, and RNA samples with a minimum RNA Integrity Number (RIN) >8 were used.

Four biological replicates were assayed. Total RNA was converted to cRNA with Cy3 or Cy5 fluorophores by means of the Low Input Quick Amp Labeling Two Color kit (Agilent). Two technical replicates were generated with dye-swap of Cy3 and Cy5 fluorophores for each condition studied. Hybridization was performed according to Agilent's instructions for two-color microarray. Slides were scanned on the SureScan Microarray Scanner (Agilent) with a resolution of 2 µm. Data extraction was performed with Feature Extraction software (Agilent) and the ISPosAndSignif flag was used to filter low intensity signal probes. All genes whose signals were not detected in the four replicates of at least one of the two experimental conditions were excluded. The intensity values of each transcript across the arrays were normalized by the quantile method [6]. For genes with multiple probes, the mean intensity among all probes showing significant change was used to represent the intensity of that gene.

Significance Analysis of Microarrays (SAM) statistical test [7] was used with cutoff q-value $\leq$0.01 (i.e., a False Discovery Rate $\leq$1%) [8]. Genes with q-value $\leq$0.01 and |log2fold-change| >1 were

considered significantly differentially expressed. The z-score was calculated for each gene in each sample, and it represents the number of standard deviations below or above the mean intensity of that gene across the various conditions that were compared; a z-scores non-supervised hierarchical clustering heatmap was plotted with Spotfire (TIBCO).

Gene Ontology analyses were performed with DAVID [9], in order to find the statistically significant enriched ontology terms of differentially expressed genes, using the Benjamini-Hochberg corrected p-value threshold of $p < 0.05$.

Gene expression correlation in the TCGA dataset

The TCGA prostate adenocarcinoma (TCGA-PRAD) dataset was used and *PVT1*-related disease-free survival analysis was done with TANRIC tool [10]. To test the 121-gene-set as a tumor risk classifier, we implemented a machine learning Random Forest algorithm in python Scikit-Learn (v.0.20.2) [11] with gene expression data from all TCGA-PRAD tumors as input.

Thus, the legacy level 3 data of Prostate adenocarcinoma (PRAD) from The Cancer Genome Atlas (TCGA) cohort were obtained from Firehose (http://gdac.broadinstitute.org). The SurvExpress [12] webtool was used to automatically convert gene IDs annotated in the Agilent microarrays for the 160 genes of interest (that were de-repressed by *PVT1* knockdown in LNCaP cells) into the gene IDs of the TCGA-PRAD RNA-seq data. Out of the 160 genes, a total of 121 were retrieved by SurvExpress in the TCGA-PRAD dataset (Supplementary Table S5). Normalized RNA-seq data of 499 TCGA-PRAD samples ("illuminahiseq_rnaseqv2-RSEM_genes_normalized") and clinical classification were used for further analysis.

In order to create a predictive profile using the 121 genes that were up regulated in LNCaP after *PVT1* silencing, we divided the TCGA prostate cancer samples in two different groups (classes), namely class 0 = intermediate-risk tumors, and class 1 = high-risk tumors. This separation was created using the National Comprehensive Cancer Network (NCCN) criteria. Intermediate risk: PSA is between 10 and 20 ng/mL, Gleason score of 7 and Classification from the TNM system T2b or T2c. High-risk: PSA > 20 ng/mL, Gleason score of 8 to 10, Classification from the TNM system T3a or T3b or T4. The final dataset is comprised of a total of 293 samples, where 119 samples represent intermediate risk tumors, and 174 high risk tumors.

The normalized expression levels of the 121 genes were used as features in a Random Forest machine learning classification model with the number of trees equal to 10k and the maximum depth 5 (the maximum number of nodes in a decision tree). The Gini Impurity metric (Gini importance score) was used to construct the trees [13]. The model was trained and evaluated using the 5-cross-validation approach, where 5 different models were created using for each model an 80%/20% (train/validation) ratio. Each of the 5 validation runs was comprised of a set of different patient samples.

The classification performance of the model was estimated by calculating the ROC area under the curve that was generated by plotting at various classification threshold settings the False Positive Rate (X-axis) and the True Positive Rate (TPR) (Y-axis), as given by the following equations:

$$TPR = \frac{TP}{TP + FN}$$

$$FPR = \frac{FP}{FP + TN}$$

Where, TP = true positives, TN = true negatives, FP = false positives and FN= false negatives.

After training the models, we also extracted the most predictive top 10 features (genes). This procedure was performed by averaging the Gini Impurity decrease between the two different groups for each feature, in the 5 different models. Intuitively, this measurement can estimate how well the intermediate-risk and high-risk tumors can be separated when a specific feature (gene) is used to create tree leaves.

The Random Forest model, Feature extraction and 5-cross-validation analyses were performed using the python Scikit-Learn (Version 0.20.2) library [11].

References

1. Zhao J, Ohsumi TK, Kung JT, Ogawa Y, Grau DJ, Sarma K, Song JJ, Kingston RE, Borowsky M, Lee JT: **Genome-wide identification of polycomb-associated RNAs by RIP-seq.** *Mol Cell* 2010, **40:**939-953.
2. O'Reilly D, Dienstbier M, Cowley SA, Vazquez P, Drozdz M, Taylor S, James WS, Murphy S: **Differentially expressed, variant U1 snRNAs regulate gene expression in human cells.** *Genome Res* 2013, **23:**281-291.
3. Koressaar T, Remm M: **Enhancements and modifications of primer design program Primer3.** *Bioinformatics* 2007, **23:**1289-1291.
4. Ayupe AC, Tahira AC, Camargo L, Beckedorff FC, Verjovski-Almeida S, Reis EM: **Global analysis of biogenesis, stability and sub-cellular localization of lncRNAs mapping to intragenic regions of the human genome.** *RNA Biol* 2015, **12:**877-892.
5. Yu Y, Zhang M, Liu J, Xu B, Yang J, Wang N, Yan S, Wang F, He X, Ji G, et al: **Long Non-coding RNA PVT1 Promotes Cell Proliferation and Migration by Silencing ANGPTL4 Expression in Cholangiocarcinoma.** *Mol Ther Nucleic Acids* 2018, **13:**503-513.
6. Bolstad BM, Irizarry RA, Astrand M, Speed TP: **A comparison of normalization methods for high density oligonucleotide array data based on variance and bias.** *Bioinformatics* 2003, **19:**185-193.
7. Tusher VG, Tibshirani R, Chu G: **Significance analysis of microarrays applied to the ionizing radiation response.** *Proc Natl Acad Sci U S A* 2001, **98:**5116-5121.
8. Benjamini Y, Hochberg Y: **Controlling the False Discovery Rate - a Practical and Powerful Approach to Multiple Testing.** *Journal of the Royal Statistical Society Series B-Statistical Methodology* 1995, **57:**289-300.
9. Huang da W, Sherman BT, Lempicki RA: **Systematic and integrative analysis of large gene lists using DAVID bioinformatics resources.** *Nat Protoc* 2009, **4:**44-57.

10. Li J, Han L, Roebuck P, Diao L, Liu L, Yuan Y, Weinstein JN, Liang H: **TANRIC: An Interactive Open Platform to Explore the Function of lncRNAs in Cancer.** *Cancer Res* 2015, **75:**3728-3737.

11. Pedregosa F, Varoquaux G, Gramfort A, Michel V, Thirion B, Grisel O, Blondel M, Prettenhofer P, Weiss R, Dubourg V, et al: **Scikit-learn: Machine Learning in Python.** *Journal of Machine Learning Research* 2012, **12:**2825-2830.

12. Aguirre-Gamboa R, Gomez-Rueda H, Martinez-Ledesma E, Martinez-Torteya A, Chacolla-Huaringa R, Rodriguez-Barrientos A, Tamez-Pena JG, Trevino V: **SurvExpress: an online biomarker validation tool and database for cancer gene expression data using survival analysis.** *PLoS One* 2013, **8:**e74250.

13. Breiman L, Friedman J, Stone CJ, Olshen RA: *Classification and regression trees.* Belmont, Calif.: Wadsworth International Group; 1984.
