# Supplementary material for: PVT1 signals an androgen-dependent transcriptional repression program in prostate cancer cells and a set of the repressed genes predicts high-risk tumors
Source: Cell Commun Signal. 2021 Jan 11;19:5. doi: 10.1186/s12964-020-00691-x (PMC7798249; doi:10.1186/s12964-020-00691-x)
Supplement: Supplementary file 3 — Additional file 2: Supplementary Figures and Tables. [file 12964_2020_691_MOESM3_ESM.pdf]

PVT1 signals an androgen-dependent transcriptional repression program in prostate cancer cells and a set of the repressed genes predicts high-risk tumors

Alexandre Videira[1,2,#], Felipe C. Beckedorff[1,2,a,#], Lucas F. daSilva[1,2,a], Sergio Verjovski-Almeida[1,2]

[1]Laboratório de Expressão Gênica em Eucariotos, Instituto Butantan, 05503-900 São Paulo, SP, Brazil.

[2]Departamento de Bioquímica, Instituto de Química, Universidade de São Paulo, 05508-900 São Paulo, SP, Brazil

# These authors have contributed equally to this work

[a]**Present address:** Sylvester Comprehensive Cancer Center, University of Miami Miller School of Medicine, Miami, Florida, USA

Supplementary Figures and Tables

Supplementary Figures S1 to S6 are given below, on pages 2 to 8.

Supplementary Table S1 is on pages 9-10, Supplementary Table S2 is on pages 11-16, Supplementary Table S3 is on pages 17-20, Supplementary Table S4 is on pages 21-23, and Supplementary Table S5 is on page 24.

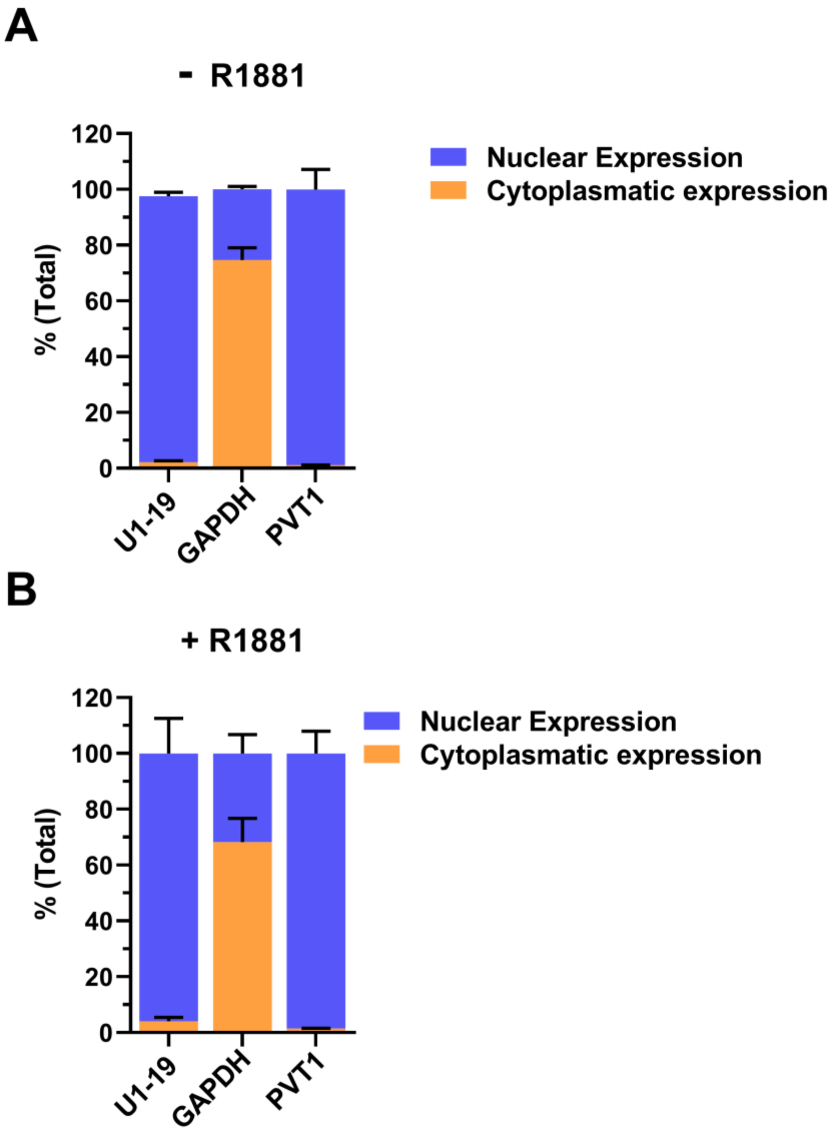

Supplementary Fig. S1. Subcellular localization of *PVT1* lincRNA in LNCaP cells. LNCaP cells in culture (**A**) with no androgen (– R1881), or (**B**) with 10 nM R1881 (+ R1881) were processed for subcellular fractionation by differential centrifugation. After nuclear and cytosolic fractions separation, RNA was extracted and the expression levels of *PVT1* in the fractions was measured by Real-time RT-qPCR. *GAPDH* was used as a cytosolic marker, and *snRNA U1-19* was used as a nuclear marker. For each gene, the % of total expression detected in each compartment is shown. Three biological replicates of each condition, with three technical replicates of qPCR per sample were assayed. Mean ± S.D. is shown.

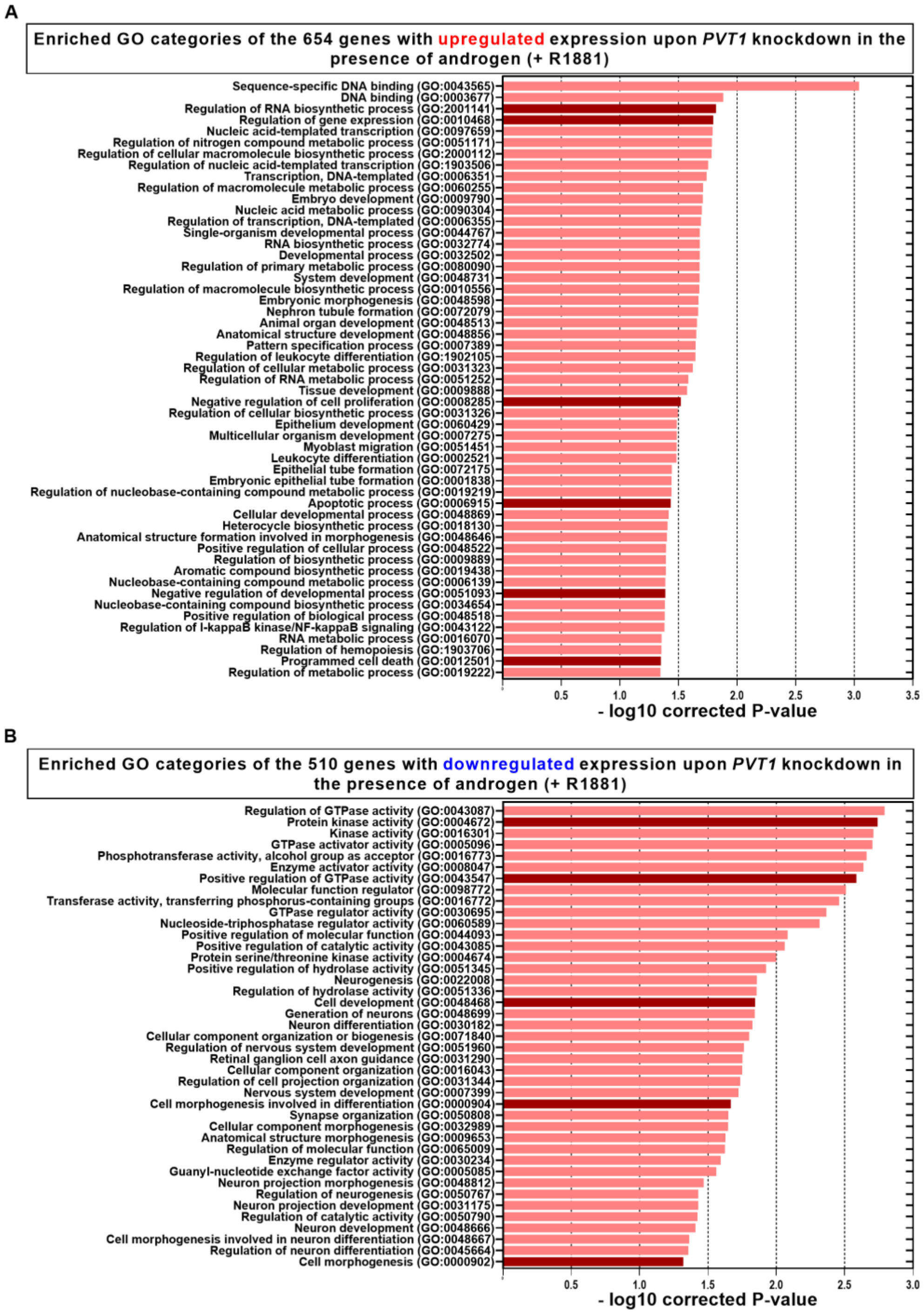

Supplementary Fig. S2. Gene Ontology (GO) terms significantly enriched among the genes whose expression was affected by *PVT1* knockdown in androgen treated LNCaP cells. Benjamini-Hochberg corrected P-values are shown on the x-axis.

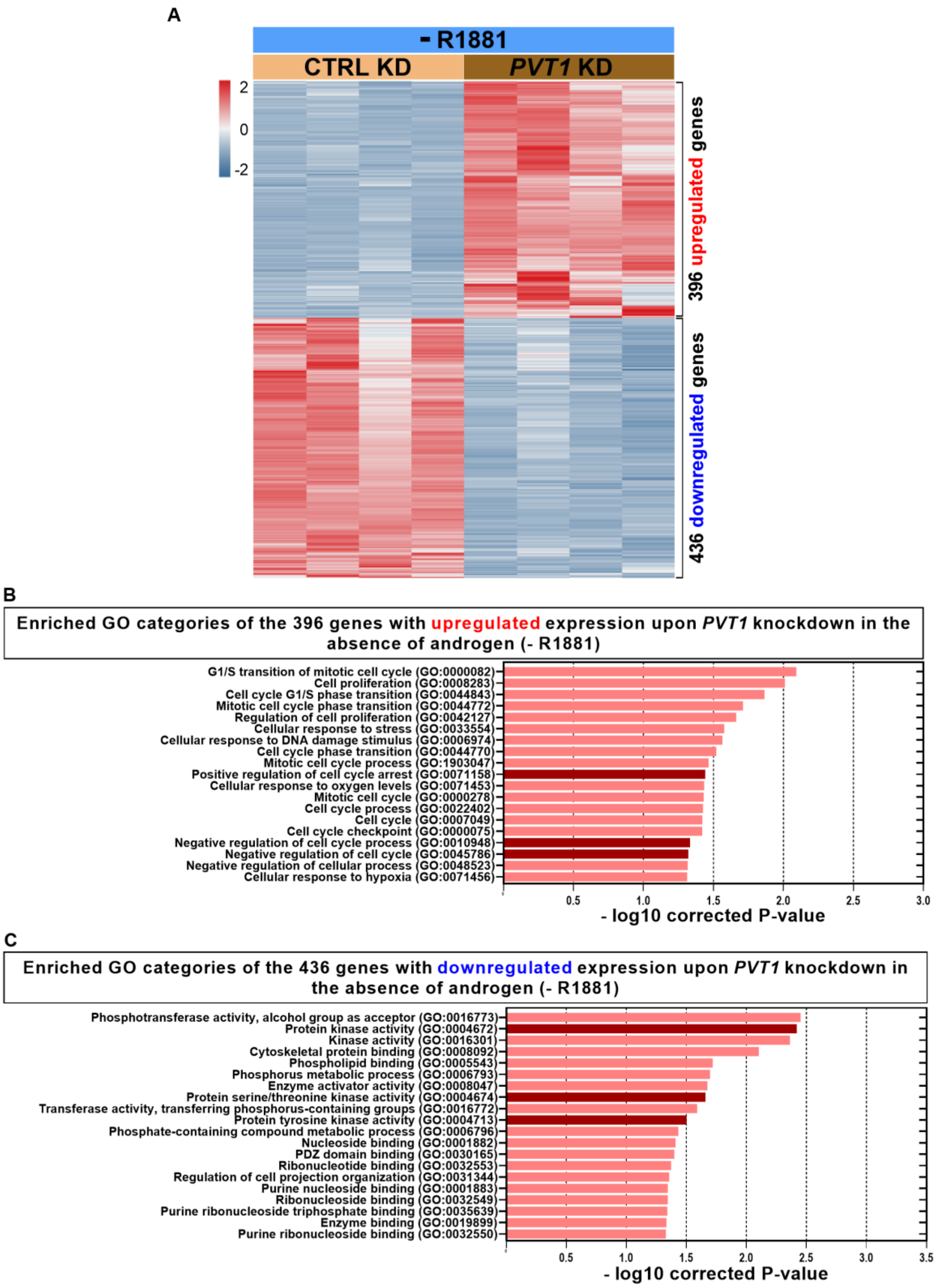

Supplementary Fig. S3. ***PVT1*** **knockdown affects the expression of hundreds of genes in hormone-starved LNCaP cells.** LNCaP cells were starved of androgen for 48 h (-R1881), then a pool

of antisense LNA GapmeR oligos targeting *PVT1* (*PVT1* KD) or a scrambled GapmeR oligo (CTRL KD) were added, cells were incubated for 24 h, followed by addition of control vehicle (ethanol) and incubation for another 24 h. RNA was extracted from four biological replicates of each condition, purified and used for gene expression measurements. **A**, detection with microarrays of genome-wide gene expression changes induced by *PVT1* knockdown (one replicate in each column, one gene in each line; z-core color scale on the left) (q-value<0.01, fold-change<0.5 or fold-change>2). **B and C,** Gene Ontology (GO) terms significantly enriched among the genes whose expression was affected by *PVT1* knockdown in hormone-starved LNCaP cells. Benjamini-Hochberg corrected P-values are shown on the x-axis. GO terms mentioned in the main text are shown with a darker red color bar.

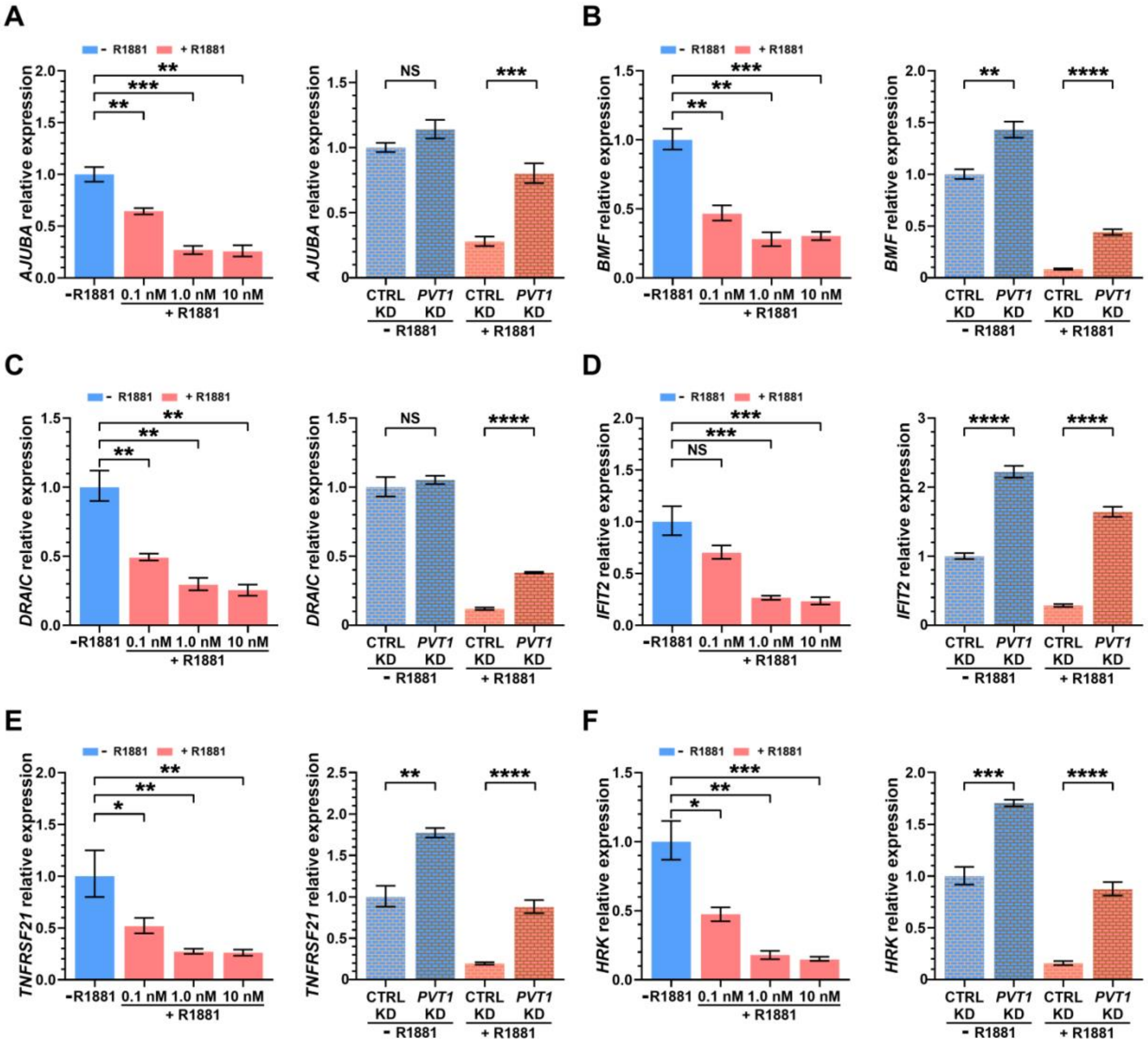

Supplementary Fig. S4. Validation by RT-qPCR of genes that were repressed by androgen and de-repressed by *PVT1* knockdown. A to F, RT-qPCR relative expression measurements of six selected tumor suppressor target genes, whose names are indicated in the Y-axis. In LNCaP cells, gene expression was repressed by androgen (+R1881) in a dose-dependent manner in the presence of endogenous *PVT1* (left part of each set from A to F), and *PVT1* knockdown (right part of each set from A to F) partially or totally restored the expression of those genes in hormone-starved cells (*PVT1* KD, -R1881, blue bars) or in androgen-treated cells (*PVT1* KD, +R1881, red bars). T-test, *P<0.05, **P<0.01, ***P<0.005, ****P<0.001; mean ± s.e.m., three biological replicates for androgen dose-dependence assays, and four biological replicates for *PVT1* knockdown assays.

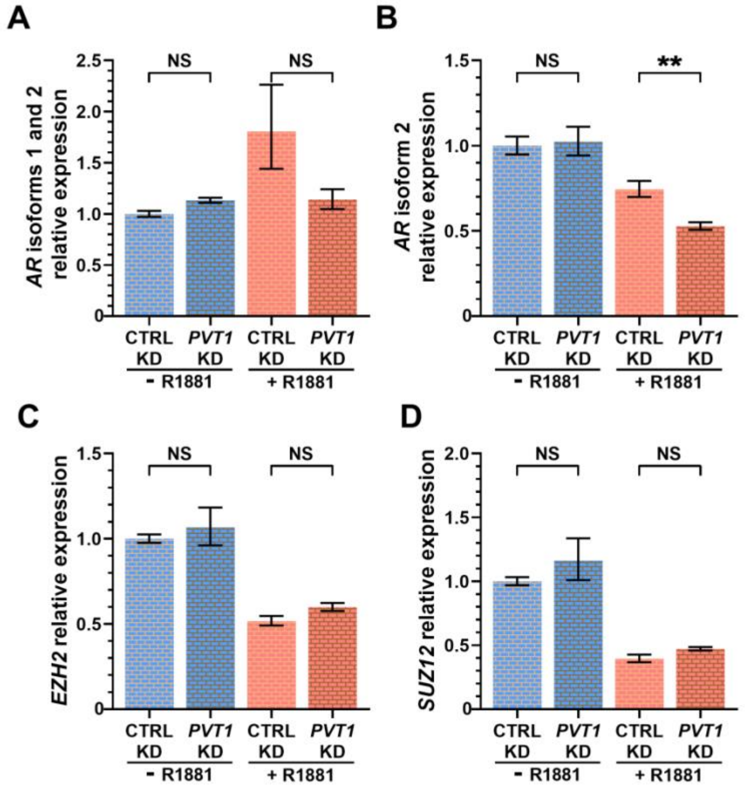

Supplementary Fig. S5. Expression level of the *AR, EZH2* and *SUZ12* genes in LNCaP cells submitted to *PVT1* knockdown. Expression levels were measured by RT-qPCR. **A**, *AR* isoforms 1 and 2 simultaneously measured. **B**, *AR* isoform 2. **C**, *EZH2*. **D**, *SUZ12*. LNCaP cells were submitted to *PVT1* knockdown (*PVT1* KD) or to control knockdown with a scrambled oligo (CTRL KD) in hormone-starved cells (-R1881, blue bars) or in androgen-treated cells (+R1881, red bars). Relative expression is shown in relation to the expression in hormone-starved cells with control knockdown. T-test, **P<0.01; mean ± s.e.m., four biological replicates.

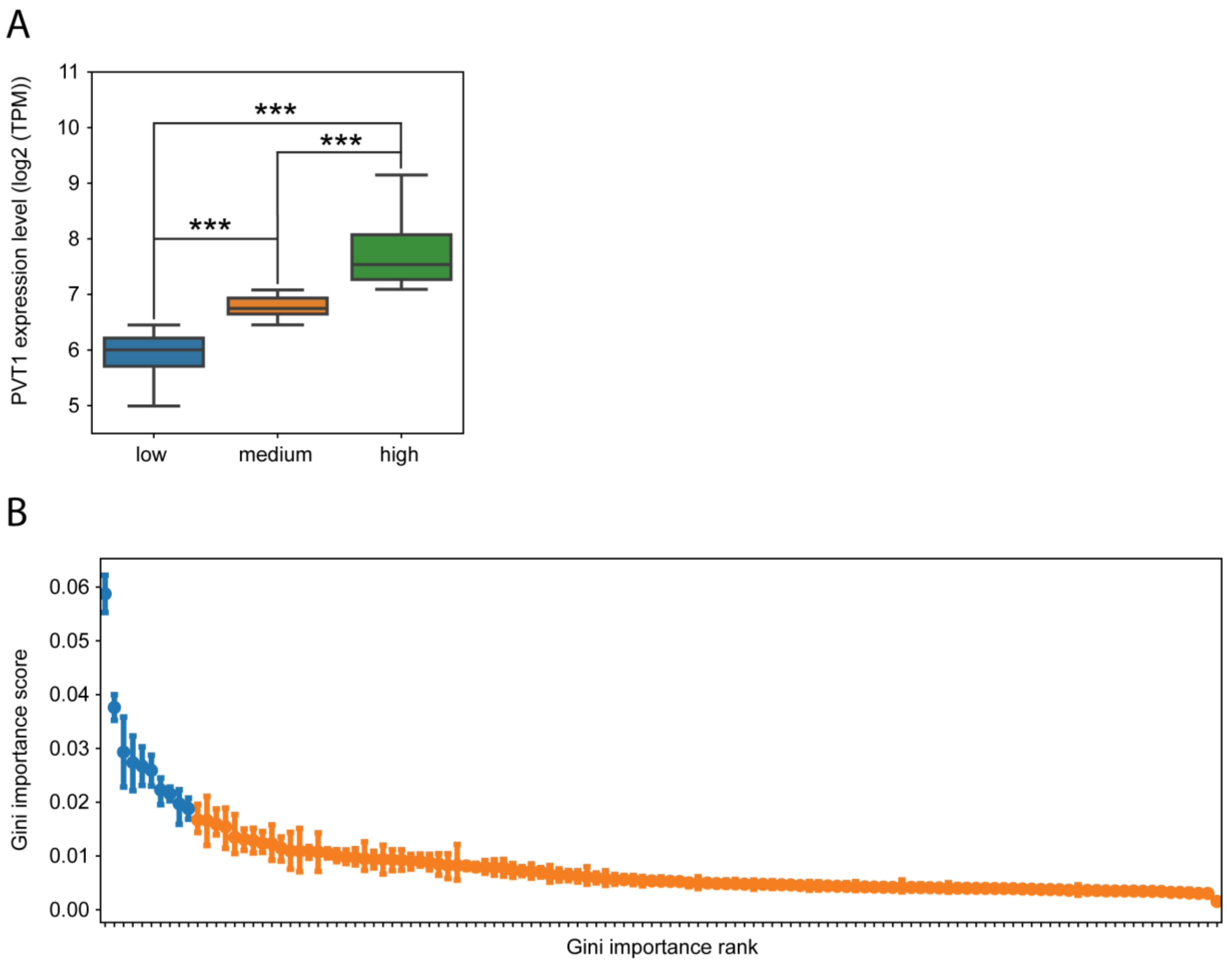

Supplementary Fig. S6. *PVT1* expression level in the TCGA-PRAD samples and Gini importance score of all genes from the 121-gene-set used in the machine-learning model for tumor-risk classification. A, Boxplot showing the *PVT1* expression levels in the tumors of 499 TCGA-PRAD patients divided into 3-quantile groups comprised of low-*PVT1* samples (blue), medium-*PVT1* (orange) and high-*PVT1* samples (green). Significance was calculated using a two-tail t-test, ***P<0.001. **B,** Gini importance score for each of the 121 genes that were upregulated after *PVT1* depletion in LNCaP cells, calculated by the Random Forest machine-learning algorithm in the TCGA-PRAD tumor-risk classification analysis. Blue points and error bars represent the Gini score mean ± s.d. (calculated from the 5-cross-validation step) for the top 10 genes with the highest Gini importance score, and orange points and error bars represent the remaining genes.

Supplementary Table S1. Oligonucleotides used in this work

Target gene	Name of oligonucleotide	Sequence 5' - 3'
**Assay: *PVT1* knockdown**		
PVT1	PVT1_2 (Exiqon LG00177218-DDA)	+A*+G*+T*G*T*C*C*T*G*G*C*A*G*+T*+A*+A
PVT1	PVT1_5 (Exiqon LG00177218-DDA)	+A*+T*+C*G*T*A*A*T*G*G*G*T*T*+G*+A*+A
Scrambled negative control	Exiqon LG00000002	
In the above sequences, the asterisk indicates a phosphorothioate linkage in the backbone of the oligonucleotide, and the "+" signal indicates a locked nucleic acid (LNA) in place of the common nucleotide.		
**Assay: RT-qPCR**		
ACTB	actb_forward	CTTCCTTCCTGGGCATGG
	actb_reverse	AGACAGCACTGTGTTGGCGTA
AJUBA	ajuba_forward	GGACCGGGATTATCACTTTGAG
	ajuba_reverse	CAACCATGGCAGAGCAAGTG
AR	ar_iso1_forward	TCAAGGGAGGTTACACCAAAGG
	ar_iso1_reverse	GAGACAGGGTAGACGGCAGTTC
	ar_iso2_forward	GCACCTTATGTCCTCCCTTCAG
	ar_iso2_reverse	CTGTGAACATGCCCAAAAGAAG
BMF	bmf_forward	TACTCAAGCGGGACCTTCTTTC
	bmf_reverse	TCACCATCACAGACACATCAGC
DRAIC	draic_forward	TGAACTCAACTCCTGAGAAGGAC
	draic_reverse	CGCTCTCAGACTCTTCAGTTCTCC
EZH2	ezh2_forward	AGTGATAGGGAAGCAGGGACTG
	ezh2_reverse	GGAGGTTCAATATTTGGCTTCATC
FAS	fas_forward	AAGGCTTTGTTCGAAAGAATGG
	fas_reverse	GATGCCAATTACGAAGCAGTTG
FASN	fasn_forward	AGGATCACAGGGACAACCTG
	fasn_reverse	ACTCCACAGGTGGGAACAAG
GAPDH	gapdh_forward	CTCTCTGCTCCTCCTGTTCGA
	gapdh_reverse	ACGACCAAATCCGTTGACTCC
HRK	hrk_forward	GCACTTCGAGAAGGAAGTGGAG
	hrk_reverse	TCTGTTTCTGCAGCTGGATTTC
IFIT2	ifit2_forward	CGGTTAAAGTGTGGAGGAAACC
	ifit2_reverse	CAGTTGTCCAGACGGTAGCTTG
NDRG1	ndrg1_forward	ACCTGCTACAACCCCCTCTT
	ndrg1_reverse	TGATCCATGGAGGGGTACAT
NOV	nov_forward	GAACCGTCAATGTGAGATGCTG
	nov_reverse	GTTCTTGAACTGCAGGTGGATG

OPRK1	oprk1_forward	ATCAATATCTGCATCTGGCTGCT
	oprk1_reverse	TAGTCATCATCTGGGAACTGCAA
PCGEM	pcgem_forward	ATCATGAGGCATTTCAGAGTGC
	pcgem_reverse	AAGGCACCTTTGTTTTCTCCAG
PRCN1	prcn1_forward	TGGAAAGGTGTCAGTGAGCAAG
	prcn1_reverse	AATATCAGCCCTTGGAATCTGG
PSA	psa_forward	GATGCTGTGAAGGTCATGGA
	psa_reverse	TGGAGGTCCACACACTGAAG
PVT1	pvt1_forward	TGGAATGTAAGACCCCGACTCT
	pvt1_reverse	GATGGCTGTATGTGCCAAGGT
SFPQ1	sfpq1_iso1_forward	TCGTACTGTTAGGCCCTTGG
	sfpq1_iso1_reverse	AACCTTGCATGAAGAGCACC
SFPQ1	sfpq1_iso2_forward	AAAACTTGCCCAGAAGAATCCA
	sfpq1_iso2_reverse	CCCTTTGCTGTTTTTCCATTTC
SI	si_forward	TTTTGGCAGCCTTATCCAAG
	si_reverse	CAATCAGAGAGATTTCCAATCCA
SUZ12	suz12_forward	TGACTCGTCCAGGAAGAAGAGAG
	suz12_reverse	CAAATGTCTTTTCCCCATCCTC
TMPRSS2	tmprss2_forward	CTGGTGGCTGATAGGGGAT
	tmprss2_reverse	GTCTGCCCTCATTTGTCGAT
TNFRSF21	tnfrsf21_forward	TTCCTCTTGGCCTTGCTCTTAG
	tnfrsf21_reverse	TGTCATCAAAGATAGGCTGCAAG
U1-19	u1_forward	CCCCACTACCACAAATTATGCAG
	u1_reverse	AAACGAAGGTGGTTTTTCTCAGG
**Assay: ChIP-qPCR**		
NOV	nov_enhancer_forward	ATGCACGTGCGTGTAAACAG
	nov_enhancer_reverse	CACAAGGTTTCTGGGTAGGG
	nov_promoter_forward	GCTGAGTGGTTTCTCCTTGTC
	nov_promoter_reverse	ACACCAGACAGCATGAGCAG
PSA	psa_enhancer_forward	GCCTGGATCTGAGAGAGATATCATC
	psa_enhancer_reverse	ACACCTTTTTTTTTCTGGATTGTTG
	psa_promoter_forward	CCTAGATGAAGTCTCCATGAGCTACA
	psa_promoter_reverse	GGGAGGGAGAGCTAGCACTTG

Supplementary Table S2. List of androgen-responsive genes in LNCaP cells. 1155 genes had their expression significantly downregulated in androgen treated cells (+R1881) compared with the expression in hormone-deprived cells (-R1881), and 883 genes showed significantly upregulated expression in androgen treated cells (+R1881) compared with the expression in hormone-deprived cells (-R1881).

**883 genes with significantly upregulated expression in androgen treated cells (+R1881)**
A_21_P0014882; A_33_P3210561; A_33_P3221980; A_33_P3298980; A_33_P3301445; A_33_P3329740; A_33_P3330413; A_33_P3349727; A_33_P3365845; A_33_P3367970; A_33_P3371450; A_33_P3375026; A_33_P3391120; A_33_P3402086; A_33_P3403356; A_33_P3407049; A1BG; ABCC1; ABCC4; ABHD2; ABHD3; ABL1; AC003101.1; AC006483.5; AC133680.1; AC142381.1; ACACA; ACAD8; ACOX3; ACSL3; ACTL8; ADAMTS1; ADH1C; ADPRM; ADRA2A; ADRB1; AF086546; AF130105; AFF3; AGPAT5; AGR2; AHNAK; AK096255; AK130248; AK4; AKAP1; AKAP12; AKAP5; AL022344.7; AL133645; ALDH1A3; ALDH4A1; ANK1; ANKH; ANKRD26P3; ANKRD37; ANXA1; AP000525.9; AP004550.1; APLN; APPBP2; ARAP2; ARG2; ARHGAP26; ARHGAP28; ARMC12; ARSG; ASGR2; ASRGL1; ATAD2; ATG101; ATMIN; ATP10A; ATP1A1; ATP1A4; ATP2C1; AZGP1; B2M; B3GNT2; BAIAP2; BC018676; BC022164; BC028196; BC029496; BC045716; BC066989; BC071853; BCAP29; BEND4; BET1; BHLHA15; BHLHE40; BICD2; BMPR1A; BMPR1B-AS1; BTAF1; BTG1; BU171032; C19orf48; C19orf83; C1orf116; C1orf21; C1QTNF9B-AS1; C2orf27A; C2orf76; C3orf35; C3orf58; C9orf152; C9orf72; C9orf91; CA5BP1; CACNG4; CAMKK2; CAND1.11; CANT1; CARTPT; CASC10; CBLL1; CBWD5; CCDC126; CCDC138; CCDC141; CCDC15; CCDC189; CCDC74A; CCDC74B; CD518305; CDC14B; CDC23; CDC25A; CDC42EP2; CDC42SE1; CDK2; CDYL2; CECR6; CENPN; CEP44; CEP83; CERS6; CHAC2; CHD7; CHKA; CHRDL1; CHRFAM7A; CITED1; CLGN; CMC2; CNGB3; COL12A1; COL23A1; CORO2A; COTL1; CREB3L2; CREB3L4; CRISPLD2; CRLS1; CROT; CSNK1A1L; CSNK1G3; CSRP2; CTD-2292M16.8; CTD-2314B22.1; CTD-2538C1.2; CTF1; CU692082; CUX2; CXCR4; CYP11A1; CYP1A1; CYP2U1; CYP4F30P; CYTH1; DA197111; DBI; DDIAS; DEFB132; DEGS1; DEPDC1B; DFNB31; DHCR24; DHRS12; DIO2; DKFZP586I1420; DLX1; DLX2; DNAJB9; DNAJC3; DNASE2B; DNM1L; DOCK4; DOCK5; DRD2; DSC1; DSG1-AS1; DUBR; DUSP2; DYNLL2; EAF2; EBP; ECI2; EDEM1; EDEM3; EFCAB12; EFNA5; EFNB2; EGFR; EHBP1; EIF4E2; ELK4; ELL2; ELOVL1; ELOVL2; ELOVL5; ELOVL7; EML1; EMP2; ENDOD1; ENST00000613924; ENST00000614063; ENST00000619210; ENST00000620237; ENST00000624585; ENST00000624615; ENTPD7; EPDR1; EPHA7; EPN2; ERGIC2; ERLEC1; ERN1; ERO1L; ERRFI1; ESRP2; ETV1; EXTL2; FAM103A1; FAM105A; FAM13A-AS1; FAM151B; FAM174B; FAM188B; FAM189A2; FAM83A-AS1; FAM91A1; FAR2P1; FAR2P2; FASN; FAXDC2; FBXW2; FCN2; FERMT2; FGD4; FGFR2; FGFR3; FGFRL1; FHDC1; FICD; FIG4; FKBP1B; FKBP5; FMO5; FOS; FOSL2; FOXD4; FRK; FRYL; FXYD3; FZD5; FZD8; GABPB1; GABPB1-AS1; GADD45G; GAN; GAPLINC; GAS8-AS1; GCFC2; GCNT1; GFM1; GHR; GLB1L2; GLI1; GLRX2; GLUD1; GLUD2; GLYATL1; GLYATL2; GMPPB; GNB4; GNMT; GOLGA2; GOLGA4; GOLPH3; GPC6; GPR158; GREB1; GRHL2; GRIN3A; GUCY1A3; H2AFZ; HACD2; HCLS1; HEBP2; HERC4; HERC5; HERC6; HERPUD1; HES2; HEY1; HGD; HIF1A-AS2; HIPK2; HK2; HM13; HMGCR; HMGCS1; HMGXB3; HOMER2; HOPX; HS3ST1; HSD17B2; HSD17B4; HSPA13; ID1; ID2; ID3; IDH1; IDI1; IDI2-AS1; IGF1; IGF1R; IGFBP3; IGFBP5; IL6R; IMPDH1; IMPDH1P11; INPP4B; INSIG1; INTS10; IRS2; ITGAV; JHDM1D-AS1; JMJD1C-AS1; JPH1; KANK1; KAZALD1; KBTBD11-OT1; KCND2; KCNG3; KCNMA1; KCNN2; KCTD3; KCTD9; KDELR2; KDM7A; KIF22; KLB; KLF4;

KLF5; KLHL29; KLK15; KLK2; KLK3; KLK4; KLK5; KLKP1; KMT5A; KRT18; KRT18P19; KRT18P26; KRT18P28; KRT18P40; KRT18P42; KRT18P49; KRT18P52; KRT18P55; KRT18P59; KRT18P64; KRT18P65; KRT19; KRT19P2; KRT8; KRT83; KRT8P15; KRT8P37; KRT8P44; KRT8P48; L3MBTL3; LA16c-380H5.4; LA16c-83F12.6; LAMA1; LAMA3; LAMC1; LAT2; LDLR; LECT1; LIFR; LIMCH1; LIN7B; LINC00644; LINC00883; LINC01296; LINC01299; LINC-PINT; lnc-AC016251.1-9:2; lnc-AGGF1-3:17; lnc-ANKH-1:3; lnc-C14orf23-3:1; lnc-C16orf61-2:1; lnc-C9orf82-1:1; lnc-CBLL1-1:3; lnc-CETP-1:1; lnc-CETP-1:2; lnc-COX4NB-1:1; lnc-DRGX-1:1; lnc-FAM75A6-1:1; lnc-GLYATL2-2:3; lnc-HDAC9-3:1; lnc-HPCAL1-1:6; lnc-KCNE4-1:1; lnc-KLRG2-1:1; lnc-KRT80-3:1; lnc-MAGEB10-2:1; lnc-MIXL1-2:1; lnc-MRPL48-1:1; lnc-MUC20-3:1; lnc-OSBPL9-1:1; lnc-PEA15-1:1; lnc-PERP-2:2; lnc-PIK3R1-1:1; lnc-PPM1D-1:1; lnc-RAB4A-1:2; lnc-RASL10A-1:1; lnc-SCCPDH-1:2; lnc-SLC7A6OS-2:1; lnc-ST3GAL5-1:1; lnc-TBX15-2:1; lnc-TSKU-1:1; lnc-WISP1-1:1; lnc-XRCC2-4:1; LOC100506023; LOC100506860; LOC100508046; LOC101927322; LOC101927482; LOC101927495; LOC158435; LOC283194; LOC284648; LOC284825; LOC440040; LOC440896; LOC440910; LOC642426; LOC81691; LONRF1; LRCH1; LRIG1; LRRC16A; LRRC8A; LRRFIP2; LSS; LY9; MAF; MAFK; MALT1; MAP2K4; MAP7D1; MAP9; MAPK10; MAPK6; MARCH5; MBOAT2; MCCC2; MCEE; MCFD2; MDGA1; MEGF9; MEMO1; MERTK; MESP1; MFSD2A; MICAL1; MICALCL; MINOS1-NBL1; MIPEP; MIR17HG; MIRLET7BHG; MLPH; MORC4; MORN1; MPC2; MPHOSPH9; MPP6; MPRIP; MPZL1; MRPS18A; MTCL1; MTERF4; MTFP1; MTM1; MTMR11; MTMR12; MTMR2; MTMR9; MTOR; MXD1; MYBL1; MYOF; MZT2A; NANS; NAT1; NAT2; NCAPD3; NDFIP2; NDRG1; NDRG4; NEBL; NEDD4L; NFIB; NFKBIA; NKX3-1; NKX3-2; NM_001098516; NM_001199814; NM_031953; NMD3; NNMT; NPC1; NPPC; NPR1; NR5A2; NSMAF; NT5DC3; NTNG1; NUP58; NUP98; NUPL1; ODC1; OLA1; OPN1MW; OR7E47P; ORC5; ORM1; ORM2; OSBPL8; OTUD7B; OTULIN; PACS1; PACSIN2; PAK1IP1; PALMD; PAPSS1; PAQR4; PAQR6; PART1; PARVA; PASK; PCDH1; PCDH20; PCNA-AS1; PCTP; PDIA5; PDLIM5; PDZRN3; PEA15; PER1; PEX10; PFKFB2; PGC; PGM2; PGM3; PHF20L1; PHGR1; PI4K2B; PIAS1; PIGH; PLA2G5; PLPP1; PLPP3; PLPP6; PMEPA1; PNKD; PNMA1; POLR3E; POP1; POTEB3; POTED; POTEI; POTEM; PPFIA3; PPFIBP1; PPFIBP2; PPM1A; PPM1D; PPM1K; PPP1CB; PPP2CB; PRAMEF8; PRDM1; PRDX6; PRIM2; PRKAR2A; PRKAR2B; PRKCA; PRKCH; PRR16; PSD3; PSMA6; PTCRA; PTGER4; PTGFR; PTN; PTPLB; PTPN21; PTPN9; PTPRJ; PTPRM; PTPRN2; RAB27A; RAB28; RAB3B; RAB4A; RAB6C-AS1; RAD54B; RALGPS2; RALYL; RARA; RARB; RASL10A; RBM24; RDH10; REP15; REPS2; RFK; RFPL2; RFPL3S; RGS16; RHCG; RHOBTB3; RHOU; RIC8B; RLN1; RNF112; RNF138; RNF138P1; RNF150; RNF223; ROR1; RP11-1078H9.6; RP11-1079K10.3; RP11-196I18.3; RP11-236B18.2; RP11-314A15.2; RP11-356O9.1; RP11-416N13.1; RP11-456K23.1; RP11-469M7.1; RP11-48B3.4; RP11-521C20.2; RP11-561O23.7; RP11-65J21.3; RP11-738O11.9; RP11-774O3.3; RP1-90G24.6; RP4-655J12.4; RP5-1198O20.4; RPL27A; RWDD4; S100P; SACS; SAP30; SASH1; SAT1; SCAP; SDCBP; SDCBPP1; SEC11C; SEC14L2; SEC24A; SEC24B; SEC24D; SEC61G; SEMA3C; SEPP1; SERPINE2; SETBP1; SGK1; SGK223; SGK3; SH3D21; SHROOM3; SIM2; SIPA1L2; SLAIN2; SLC16A1; SLC16A6; SLC22A1; SLC22A17; SLC22A23; SLC25A20; SLC25A33; SLC25A37; SLC25A40; SLC26A2; SLC2A1; SLC30A7; SLC33A1; SLC35F2; SLC35G5; SLC39A10; SLC39A14; SLC39A7; SLC41A1; SLC43A1; SLC45A3; SLC4A4; SLCO2A1; SLF1; SLIT3; SMAD9; SMAGP; SMIM13; SMPD2; SMPD4; SMS; SNAP23; SNORA19; SNORA30; SNORA80A; SNORD90; SOCS2; SOCS2-AS1; SORD; SP9; SPATA13; SPATC1L; SPATS2L; SPCS3; SPDEF; SPDYA; SPHAR; SPRED1; SPTB; SRF; SSR3; SSX2IP; ST20-AS1; ST6GAL1; ST6GALNAC1;

STARD3NL; STK17B; STK39; SULT1C3; SUN2; SWT1; SYNJ1; SYT4; SYTL1; TACSTD2; TAF1A; TAOK3; TARP; TARS; TASP1; TBC1D1; TBC1D4; TBC1D8; TBRG1; TBX2; TBXAS1; TCAF1; TCAF2; TCONS_l2_00006720; TCONS_l2_00017540; TCONS_l2_00018295; TCONS_l2_00018296; TCONS_l2_00028266; TENM1; TEX2; TG; THC2568408; THC2610134; THC2626681; THC2636494; THC2665222; THC2685534; THC2719114; THRAP3; THRB; THSD7A; THUMPD1; THYN1; TIPARP; TM4SF1; TMCC3; TMED9; TMEFF2; TMEM100; TMEM164; TMEM79; TMEM87A; TMEM87B; TMPRSS2; TNFAIP3; TNFAIP8; TNFRSF19; TNK2; TPD52; TRAPPC11; TRG-AS1; TRIB2; TRIM24; TRIM36; TRIM48; TRIM49; TRIM49B; TRIM49D1; TRIM51; TRIM53AP; TRMT6; TRPM7; TSKU; TTC12; TTC5; TTN; TUBA3C; TUBA3D; TUBA3FP; TUBA3GP; TWIST1; TXNDC16; U71363; UAP1; UBE2G1; UBE2J1; UBE2NL; UGT1A6; UGT2B7; UHRF2; UHRF2P1; USP10; USP8; VEGFA; VGF; VIMP; VLDLR; VLDLR-AS1; VSTM2A; WDR41; WDYHV1; WIPI1; WNT7B; WTH3DI; WTIP; WWC1; XM_003959913; XR_109259; XR_109894; XR_171099; XR_242328; XR_242411; XR_424109; XR_428440; XR_433669; ZBED3-AS1; ZBTB1; ZBTB10; ZBTB16; ZBTB24; ZBTB44; ZC3HAV1; ZCCHC6; ZDHHC8P1; ZHX1; ZNF18; ZNF385B; ZNF532; ZNF652; ZNF69; ZNF814; ZNRF2; ZNRF2P1; ZP1.

**1155 genes with significantly downregulated expression in androgen treated cells (+R1881)**
A_21_P0014213; A_21_P0014894; A_32_P48615; A_33_P3222600; A_33_P3241150; A_33_P3265935; A_33_P3294078; A_33_P3311911; A_33_P3346526; A_33_P3392350; A2M-AS1; ABCA1; ABCA12; ABCC5; ABCC6; ABCC6P1; ABCG1; ABLIM1; ABTB2; AC007562.1; AC010967.2; AC073283.4; AC093901.1; AC141928.1; ACAA2; ACADSB; ACKR3; ACTA2; ACTRT3; ADAM23; ADCY1; ADCY2; ADD2; ADGRB2; ADGRG6; ADGRV1; ADM; ADORA2B; ADRA2C; AEBP1; AGBL5-AS1; AGT; AHNAK2; AIDA; AJUBA; AK021734; AK058041; AK092862; AK123308; AK124361; AK124859; AK125166; AK125979; AK129982; AKR1C1; AKR1C3; AL117431; AL523350; ALCAM; ALPK1; ALX4; AMIGO2; AMIGO3; ANGPTL2; ANKRD16; ANKRD18A; ANKRD18B; ANKRD20A11P; ANKRD20A12P; ANKRD20A2; ANKRD20A3; ANKRD20A5P; ANKRD20A8P; ANKRD20A9P; ANKRD28; ANKRD36B; ANKRD62P1; ANO2; ANP32E; ANPEP; ANTXR1; ANXA9; AOC1; AP000253.1; APBA1; APOLD1; APPL1; ARHGAP18; ARHGAP20; ARHGAP27; ARHGAP32; ARHGEF10; ARHGEF17; ARHGEF40; ARL4D; ASAP2; ASB13; ASIC1; ASNSP1; ASS1; ATG16L2; ATG3; ATOH7; ATP11A; ATP1B2; ATP2B1; ATP2B4; ATP8A1; ATXN7L1; AUTS2; BAHCC1; BAMBI; BANK1; BARD1; BASP1; BBS9; BC092495; BCAM; BCHE; BDH2; BEND5; BEND7; BEST1; BEST4; BFSP1; BHLHE41; BIRC3; BLNK; BMF; BMX; BRSK2; BTD; BTN2A2; BX648696; C10orf10; C12orf60; C14orf132; C14orf93; C16orf45; C16orf74; C16orf89; C18orf61; C1orf63; C1QTNF3; C1QTNF6; C1R; C1RL-AS1; C21orf58; C2orf15; C4orf32; C4orf46; C5orf30; C9orf92; CA12; CABLES1; CACNA1G; CACNB2; CADM1; CALD1; CAMK1D; CAMK2N1; CAPRIN2; CAPS2; CARNS1; CASC1; CASC2; CBLN2; CBR3; CCDC14; CCDC18-AS1; CCDC69; CCDC88B; CCDC93; CCNE2; CCNG2; CCNO; CD24; CD83; CDCA7L; CDH18; CDH26; CDH3; CDHR3; CDK8; CDNF; CDON; CDR2L; CELA2A; CERK; CFAP70; CHAC1; CHADL; CHAF1B; CHN1; CHRM1; CLCN4; CLCN5; CLDND2; CNBD1; CNKSR3; CNTN2; CNTN5; COBL; COBLL1; COL16A1; COL18A1; COL5A2; COL9A2; COL9A3; COLCA1; COLQ; COMP; CPAMD8; CRABP2; CREBRF; CRNDE; CROCCP2; CSMD1; CTBP1; CTD-2251F13.1; CTD-2342N23.3; CTTNBP2NL; CXorf36; CYTH3; DAND5; DAPK2; DCBLD2; DCDC2; DCHS1; DDB2; DDC; DDO; DDR2; DDX11L16; DDX58; DENND1B; DENND3;

DENND5B; DEPTOR; DFNA5; DGKH; DICER1-AS1; DIDO1; DIO3; DIXDC1; DLGAP1-AS3; DMKN; DNAH11; DNAH2; DOK1; DOK7; DPP4; DPYSL3; DQ786257; DRAIC; DSC2; DSE; DSEL; DUSP26; DUSP6; EDN2; EFCAB11; EFHD1; EFR3B; EGLN3; ELFN2; ENC1; ENOX1; ENPP4; ENPP5; ENST00000612143; ENST00000614678; ENST00000614987; ENST00000615792; ENST00000619168; ENST00000621666; ENST00000621996; ENST00000623723; ENST00000624675; ENTPD3; EPB41; EPB41L4A; EPHX1; EPS8; ERBB2; ESPN; ETV6; EXPH5; EXTL1; F2R; FALEC; FAM102A; FAM110C; FAM134B; FAM175A; FAM177B; FAM182B; FAM184A; FAM184B; FAM198A; FAM198B; FAM19A2; FAM201A; FAM212B; FAM217B; FAM221B; FAM224A; FAM229B; FAM47E; FAM49A; FAM78A; FANK1; FAS; FAXC; FBXL2; FBXL22; FBXO15; FCGR2C; FCN1; FEZ1; FGD3; FGD6; FGF13; FHOD3; FLJ31104; FLJ36000; FLJ43315; FMNL3; FOLH1; FOLH1B; FOXD3; FOXN3; FOXN4; FOXO4; FOXO6; FOXP4-AS1; FOXQ1; FRMD5; FRMPD1; FRMPD2; FSCN1; FUT10; FUT8; FUT8-AS1; FYN; FZD2; FZD3; FZD4; GALNT10; GALNT14; GALNT18; GAREM1; GATA6; GATS; GCA; GCG; GHRL; GIMAP2; GIMAP6; GJB2; GLCCI1; GLCE; GLI3; GLIPR1L2; GLIS2; GLS2; GOLGA2P2Y; GOLGA2P6; GOLGA6L4; GOLGA6L9; GOLGA8R; GOLPH3L; GPAT3; GPER1; GPR137B; GPR20; GPR63; GPRIN2; GPX8; GRAMD1C; GRB10; GRB14; GRIK1; GRIK2; GRK5; GS1-421I3.2; GSC; GSTA2; GSTA5; GUCY1B2; GUSBP1; GUSBP2; HACL1; HAGLROS; HCG8; HEG1; HEPACAM; HLA-DQB1; HOTAIRM1; HOXA10; HOXA11-AS; HOXA3; HOXA5; HOXA7; HOXA9; HOXB5; HOXB6; HOXB-AS1; HOXC12; HOXC13-AS; HOXC4; HOXC5; HOXC8; HOXC9; HOXC-AS1; HOXC-AS3; HRK; HS6ST2; HSD17B7; HSPA12A; HSPA4L; HTR2C; HTRA1; IER5; IFIH1; IFIT2; IFIT5; IFITM1; IGFALS; IGIP; IGKV1-16; IGKV1D-8; IGKV1OR2-108; IKZF2; IKZF3; IL15; IL17C; IL17RB; IL17RD; IL1RAP; IL1RN; IL27RA; IL36G; IL4R; INHBB; INSIG2; INTS8; IQCH-AS1; IQCJ-SCHIP1; IRF1; IRF2BPL; IRX3; IRX5; ITGB4; ITGB7; ITPKA; ITPR1; JAK3; JDP2; KALRN; KATNAL2; KCNAB2; KCNG1; KCNIP3; KCNJ3; KCNK13; KCNK5; KCNMB4; KCNS3; KCNU1; KDM4D; KDM6A; KHDRBS3; KHK; KIAA0408; KIAA0922; KIAA1217; KIAA1257; KIAA1324; KIAA1462; KIF21A; KIF25; KIF3C; KIF5B; KITLG; KLHL1; KLHL24; KLHL3; KLLN; KMT2C; KSR2; LAMB1; LCN15; LETMD1; LFNG; LIF; LIMA1; LIMS2; LINC00467; LINC00476; LINC00478; LINC00565; LINC00648; LINC00673; LINC00853; LINC00858; LINC00933; LINC01003; LINC01006; LINC01021; LINC01024; LINC01133; LINC01137; LINC01138; LINC01184; LINC01193; LINC01351; LINC01354; LINC01503; LINC01569; LINGO3; lnc-ACCSL-1:1; lnc-ACSS3-2:1; lnc-ACSS3-2:2; lnc-ADA-1:2; lnc-BDKRB1-1:1; lnc-BHLHE41-2:12; lnc-C11orf39-3:1; lnc-C12orf61-3:1; lnc-C1orf122-1:1; lnc-C1orf201-2:2; lnc-C1orf201-3:2; lnc-C5orf43-3:2; lnc-CCKAR-1:1; lnc-CCKAR-1:2; lnc-CCNE2-1:1; lnc-COBLL1-1:1; lnc-COLQ-1:1; lnc-CTD-2054N24.2.1-1:1; lnc-DCAF10-2:1; lnc-DHX34-2:1; lnc-DTHD1-2:1; lnc-DYDC1-3:1; lnc-EVX1-5:3; lnc-EXOC4-2:1; lnc-EYS-2:1; lnc-F8A2-2:1; lnc-FAM72C-2:1; lnc-FOXN1-1:1; lnc-FURIN-1:1; lnc-FZD4-1:1; lnc-GOLPH3L-1:1; lnc-HIATL1-1:1; lnc-INTS8-1:1; lnc-INTS9-1:3; lnc-IRF2BPL-2:1; lnc-IRX3-4:5; lnc-JMJD7-PLA2G4B-1:2; lnc-JMJD7-PLA2G4B-2:1; lnc-KB-1980E6.3.1-1:2; lnc-KDM6A-1:3; lnc-KIAA0195-1:1; lnc-KIAA0319L-1:1; lnc-LOXL1-1:1; lnc-MAT2B-3:19; lnc-MAT2B-3:8; lnc-MBL2-2:3; lnc-MDM4-1:1; lnc-MLLT4-1:1; lnc-OIT3-2:1; lnc-PDCD11-1:1; lnc-PDE2A-1:1; lnc-PGR-1:1; lnc-PHF3-1:1; lnc-PHGDH-2:1; lnc-PRAGMIN.1-3:3; lnc-QKI-5:1; lnc-RASSF7-1:1; lnc-RP11-293M10.1.1-1:1; lnc-RP3-377D14.1.1-3:12; lnc-RPLP1-1:14; lnc-RPLP1-1:15; lnc-SLC2A9-1:1; lnc-SLC38A8-4:2; lnc-SLC3A2-2:1; lnc-SRBD1-1:1; lnc-STK32C-2:1; lnc-THNSL1-2:1; lnc-TMEM140-1:1; lnc-TNFAIP8L1-1:1; lnc-TRAPPC8-2:1; lnc-TRH-2:1; lnc-TULP4-1:1; lnc-VSIG2-1:1; lnc-WASF3-1:1; lnc-ZBBX-3:1; lnc-ZNF667-2:1; lnc-ZNF674-3:2; lnc-ZNF74-1:5; LNP1; LNX2;

LOC100129203; LOC100130357; LOC100131347; LOC100288798; LOC100288911; LOC100289495; LOC100505478; LOC100506082; LOC100506476; LOC100506990; LOC100507487; LOC100996255; LOC100996455; LOC101927056; LOC101927571; LOC101927822; LOC101928103; LOC101929395; LOC101929633; LOC155060; LOC257396; LOC283575; LOC388242; LOC400958; LOC401585; LOC642929; LOC643072; LOC643770; LOC646214; LOC650293; LOC728730; LPCAT4; LRIG3; LRRC20; LRRC31; LRRC4C; LRRC75A; LRRC9; LRRN1; LTF; LTN1; LUZP2; LYN; MACF1; MAGI3; MAL; MAN1C1; MANBA; MANEA; MANEA-AS1; MAP2K6; MAP3K13; MAP3K8; MAPK4; MAPRE2; MATN2; MB21D2; MCAM; MCF2L; MCTP1; MCU; MECOM; MED14OS; MET; METTL15; MEX3B; MFI2-AS1; MGC16275; MIR4697HG; MIR600HG; MMP16; MMP24; MNX1; MNX1-AS1; MORC3; MOV10L1; MRC2; MRS2; MT1DP; MTMR9LP; MUT; MX1; MYB; MYH10; MYLK; MYNN; MYRIP; MYT1; MYZAP; NAB1; NABP1; NAIP; NANOS1; NAP1L3; NAPEPLD; NAT8L; NCOA7; NDUFAF4; NEK1; NEK6; NET1; NETO1; NEURL3; NINL; NIPAL1; NIPSNAP3A; NIPSNAP3B; NKAPP1; NKD2; NLGN4X; NLRC3; NOV; NOVA1; NPAS1; NR_024011; NR3C1; NR3C2; NR4A2; NREP; NRG3; NRIP3; NRXN1; NSG1; NTN4; NUAK1; NUAK2; NUDT8; NUTM2F; OCLN; OGFRL1; OPRK1; OPTN; OR51E1; OSBPL10; OSR2; OXTR; P2RX7; PABPC5; PAG1; PAK2; PAN3-AS1; PAPPA; PARP8; PAX1; PBX1; PBXIP1; PCDH11X; PCED1B; PDE4D; PDE4DIP; PDGFRL; PDLIM3; PDZK1IP1; PEG3; PELI2; PGM2L1; PGM5P2; PHF12; PHLPP1; PIGZ; PIK3IP1; PIK3IP1-AS1; PIK3R3; PITX1; PKIA; PKIB; PKNOX2; PLA2G2A; PLA2G4D; PLCB2; PLCB4; PLD6; PLEKHA2; PLGLB1; PLIN4; PLS1; PMP22; PMS2; PPP1R14C; PPP2R2A; PRAC2; PRDM12; PRKAG2-AS1; PRKD1; PRKG2; PRSS16; PRSS23; PSAT1; PSAT1P1; PSAT1P3; PTPRK; PTPRR; PURA; RAB17; RAB19; RAB33A; RAB37; RABL3; RAD21-AS1; RAI14; RANBP3L; RAP1GDS1; RAP2B; RAP2C-AS1; RARRES3; RASA4; RASL11B; RASSF2; RASSF5; RASSF8-AS1; RBP5; RBPMS; RCAN2; RCAN3; RDM1; REEP1; RELB; RFX3-AS1; RGL1; RGS10; RHBG; RHOH; RIMS1; RMDN1; RNASEL; RNF125; RNF144B; RNF157-AS1; RNF43; RNFT2; ROR2; RP11-1000B6.3; RP11-1023L17.1; RP11-1081M5.1; RP11-1081M5.2; RP11-119D9.1; RP11-12K11.2; RP11-253M7.4; RP11-254F7.2; RP11-267A15.1; RP11-299H21.1; RP11-319E16.2; RP11-319F12.2; RP11-329B9.4; RP11-356J5.12; RP11-379B8.1; RP11-383J24.6; RP11-401P9.4; RP11-421M1.8; RP11-450H5.1; RP11-457M11.5; RP11-45M22.5; RP11-493L12.4; RP11-499E18.1; RP11-539L10.2; RP11-578F21.6; RP11-57A19.2; RP11-629O1.2; RP11-657O9.1; RP11-666A20.4; RP11-699A5.2; RP11-725D20.1; RP11-760H22.2; RP11-77P16.4; RP11-85M11.2; RP11-888D10.3; RP11-98G7.1; RP13-726E6.1; RP13-941N14.1; RP1-65J11.1; RP4-800G7.2; RPS6KA5; RPS6KL1; RSBN1L-AS1; RTN4R; RTN4RL1; RYR2; S100A3; SCAI; SCGB1D1; SCGB1D2; SCHIP1; SCNN1D; SDAD1P1; SDC3; SDC4; SELL; SEMA5B; SEPT6; SERAC1; SERPINB5; SERPINI1; SESN3; SFR1; SH2D3C; SH3BGRL2; SH3BP5; SH3RF1; SHC3; SHC4; SHISA3; SI; SKAP2; SLC12A2; SLC12A6; SLC16A14; SLC16A7; SLC18B1; SLC1A7; SLC20A2; SLC22A10; SLC22A11; SLC23A1; SLC2A13; SLC30A3; SLC35F3; SLC38A9; SLC40A1; SLC44A1; SLC51A; SLC52A3; SLC6A12; SLC6A13; SLC7A2; SLCO5A1; SLITRK3; SMA4; SMAD3; SMC6; SMIM3; SNAI3; SNRK; SNRK-AS1; SNX18P3; SNX18P7; SNX22; SOBP; SOGA3; SORBS1; SORBS2; SORL1; SOWAHB; SOX11; SPAG5-AS1; SPTAN1; SPTLC3; SRCIN1; SRGAP2; SRGAP2B; SRGAP3; SRP14-AS1; SSBP2; ST3GAL1; ST5; ST7; ST7-AS1; ST7-OT4; ST8SIA4; STAC; STARD5; STK3; STOX1; STOX2; STRBP; STXBP5L; STXBP6; SULF2; SUSD4; SUSD6; SVIL; SYBU; SYNE1; SYNE2; SYNPO; SYTL2; TAL2; TARBP1; TARSL2; TBX10; TBX19; TCF7L2; TCONS_l2_00000198; TCONS_l2_00000562; TCONS_l2_00001498; TCONS_l2_00007046; TCONS_l2_00008795; TCONS_l2_00008799; TCONS_l2_00008879; TCONS_l2_00008962; TCONS_l2_00017138; TCONS_l2_00017422; TCONS_l2_00018853; TCONS_l2_00018976;

TCONS_l2_00019490; TCONS_l2_00020648; TCONS_l2_00020852; TCONS_l2_00021053; TCONS_l2_00022830; TCONS_l2_00022832; TCONS_l2_00023776; TCONS_l2_00028458; TCONS_l2_00028604; TCONS_l2_00028817; TCONS_l2_00029295; TCONS_l2_00029343; TCTEX1D4; TDP1; TFAP2A-AS1; TFF3; TGM3; THBS4; THC2471881; THC2507047; THC2537043; THC2548652; THC2563836; THC2567789; THC2600547; THC2601170; THC2644314; TIAM2; TIMP2; TLE1; TLE3; TLL1; TMCC2; TMEM116; TMEM117; TMEM144; TMEM158; TMEM181; TMEM266; TMEM38B; TMEM45A; TMEM51; TMPRSS6; TMSB4X; TNFRSF10D; TNFRSF11B; TNFRSF12A; TNFRSF21; TNFSF15; TNIK; TNRC6B; TNS1; TOX3; TP53INP1; TPCN1; TPPP; TPRXL; TRBV30; TRERF1; TRIB1; TRIM2; TRIM38; TRIM45; TRNP1; TRPS1; TSHZ1; TSHZ3; TSL; TSPAN10; TSPAN12; TSPAN5; TTBK1; TTBK2; TTN-AS1; TTTY14; TTTY6; TUBB8; TULP4; TYRP1; UACA; UBAC2-AS1; UBE2E1-AS1; UBE2Q2P1; UBE2Q2P3; UFL1; UGT2B10; UGT2B11; UGT2B15; UNC5B; USP3-AS1; VASH2; VAV3; VGLL4; VIPR2; VN1R108P; VOPP1; VPS13A; VPS13B; VPS54; VSTM2L; VTA1; VWA2; VWF; WASIR2; WEE2-AS1; WI2-85898F10.1; WLS; WNT4; XM_006723786; XR_109175; XR_111287; XR_158870; XR_159012; XR_241604; XR_241611; XR_242359; XR_242407; XR_242480; XR_244659; XR_244755; XR_245037; XR_245646; XR_246983; XR_250613; XR_251551; XR_251682; XR_252953; XR_424060; XR_424361; XR_424475; XR_425623; XR_425926; XR_426345; XR_426478; XR_426840; XR_431240; XR_432026; YPEL1; ZADH2; ZAK; ZBBX; ZBTB20; ZBTB43; ZC3H6; ZCWPW1; ZFHX2; ZFHX4; ZFP36L1; ZIC2; ZIC5; ZMAT1; ZMYND12; ZNF117; ZNF204P; ZNF365; ZNF575; ZNF658; ZNF703; ZNF81; ZSWIM4; ZSWIM5.

Supplementary Table S3. List of 654 genes that had a significantly upregulated expression upon *PVT1* knockdown and of 510 genes that had a significantly downregulated expression upon *PVT1* knockdown in androgen treated LNCaP cells (+R1881).

**654 genes with a significantly upregulated expression upon *PVT1* knockdown in androgen treated LNCaP cells (+R1881)**
A_33_P3223860; A_33_P3301876; A_33_P3345031; AC024560.2; ACRC; ACTA1; ADAM20P1; ADRB1; AEN; AK025118; AK096098; AK123926; AK127152; ALB; ANXA1; ANXA2R; AREG; ARL4A; ARRDC2; ASH1L-AS1; AURKAPS1; BBC3; BRD8; BRDT; BRI3; BTN3A1; BTN3A2; C1orf56; C2orf72; C2orf81; C4orf46; CARD9; CCNE2; CD3EAP; CD55; CDH26; CEACAM1; CEP135; CHAC1; CHRNA10; CLDN11; COX19; CTD-2292M16.8; CTXN1; CYP27A1; DCUN1D5; DDIAS; DHX58; DIEXF; DLX2; DNAAF3; DNHD1; DOK3; DTL; DTWD1; DUSP18; DYRK3; E2F7; ECM1; EFCAB3; EGR1; ENKUR; ERAP2; ESPL1; ESPNL; FAM155B; FAM187A; FAM209A; FAM46C; FAM71A; FAM81A; FAM84A; FAM87A; FAS; FAXDC2; FDXR; FGD3; FOXD4L5; FOXN4; FOXQ1; GADD45A; GATA6; GLS; GPRIN1; GPX8; GRM2; GSG2; GUCY1B2; HCLS1; HRK; IDI2-AS1; IER5; IFFO1; IFIT1; IL20RB; IQCD; IRS1; KCNIP2-AS1; KCNJ14; KIAA0408; KIAA1462; KIF2C; KLHL31; LAMB2P1; LIF; LINC00324; LINC00476; LINC01311; LINC01410; LMO2; lnc-AK5-1:1; lnc-AP000769.1-1:1; lnc-CERK-1:1; lnc-DCTD-1:1; lnc-DKK4-1:1; lnc-FAM72C-2:1; lnc-FAM98A-1:1; lnc-IDE-1:1; lnc-MATN2-2:1; lnc-MMRN1-2:1; lnc-MMRN1-2:2; lnc-MMRN1-2:5; lnc-NDST2-3:1; lnc-NT5DC3-1:1; lnc-OBFC2A-1:1; lnc-PITPNC1-1:1; lnc-PPM1D-1:1; lnc-RPL7L1-1:1; lnc-SEPT7L-1:1; lnc-SERPINC1-1:24; lnc-SLC39A8-1:1; lnc-SRBD1-1:1; lnc-TAF15-1:1; lnc-WNT1-2:1; lnc-WRAP73-1:1; LOC100506804; MCM10; MDM2; MESDC1; MIA; MORF4L2-AS1; MPZL2; MRM1; MS4A8; MTFR2; MTHFD2; MYB; NAB2; NABP1; NOV; NR2F1; ORC6; PAX1; PAX2; PDE4A; PDE4D; PDE4DIP; PGF; PHLDA1; PHLDA3; PIM2; PLAC8L1; PLK3; PMAIP1; PMP22; PPP1R27; PRKAG2-AS1; PTGES2-AS1; PTGFR; RASL11B; RBM26-AS1; REM2; RHBDF2; RHEBL1; RIN1; RNF19B; RP11-318C24.2; RP11-344N10.5; RP11-395G23.3; RP11-621L6.3; RPA4; RPRML; RRN3P2; SBSN; SCARNA8; SKA1; SLC18B1; SLC25A19; SLC25A36; SLC29A1; SLC7A2; SNHG1; SNHG12; SNORA26; SNORA3B; SNORA8; SNORD103A; SNORD21; SNORD43; SNORD47; SNORD84; SNORD86; SOGA3; SPG7; SPRY1; STC2; TBX2; TCONS_l2_00009373; TCONS_l2_00020648; TFAP2A-AS1; THC2667604; THC2724751; TICRR; TIGAR; TLR3; TMED6; TMEM243; TNFSF10; TNFSF9; TOR4A; TSPAN12; TTR; UBAP2; UHRF1; WDR66; XR_241982; ZC3H12C; ZFP36L2; ZGLP1; ZNF124; ZNF215; ZNF436-AS1; A_21_P0014946; A_32_P88905; A_33_P3218504; A_33_P3334043; A_33_P3346526; A_33_P3404759; A_33_P3424347; ABT1; AC092620.3; AC093382.1; AC108488.4; ACKR3; ACTA2; ADH1C; ADM; AGBL5-AS1; AJUBA; AK093004; AK095428; AK095971; AK096487; AK096649; AK097098; AK098491; AK123449; AK124361; AK126805; AK289844; AK294208; AMIGO3; AMOTL2; ANG; ANKRD16; ANKRD34A; ANKRD50; ANP32E; AQP10; ARHGAP32; ARL4D; ARMCX6; ASB13; ASNSP1; ASPM; AY129018; BAX; BBS12; BC029571; BC050402; BEX5; BF515046; BI056255; BIRC3; BMF; BRIP1; BTG2; C10orf10; C11orf95; C12orf66; C4orf47; C8orf48; CAPN10-AS1; CARD8; CBLN2; CBR3; CCDC103; CCDC34; CCNO; CD83; CDADC1; CDH24; CDKL1; CDKN1A; CEP120; CEP152; CEP162; CERK; CHKB-AS1; CHML; CLCN5; CLDN23; CLPTM1; CNKSR3; COLCA1; COLGALT1; CRYGS; CSRNP2; CTD-2319I12.4; CUZD1; CYP2J2; DAND5; DCLRE1B; DDX11L16; DESI2; DNAH11; DNMT3A; DRAIC; DRAM1; DSC1; DSC2; DUSP4; DYRK2; EEF1DP3; EID2B; EMC3-AS1; ENC1; ENST00000621052; ENST00000623723; ERICH2; ETS2; EXPH5; F2R; FAM155A-IT1;

FAM177A1; FAM198B; FAM212B; FAM217B; FAM229B; FAM50B; FGD6; FIGN; FOS; FOXD3; FOXO4; FOXO6; FRMD4B; FSCN1; FSD1L; FZD7; FZD8; GDPGP1; GEMIN2; GIGYF1; GIMAP2; GIT1; GJB2; GJC1; GNPDA1; GPAT3; GRHL3; HACD1; HAGLROS; HAS3; HAUS6; HEG1; HES6; HMBOX1; HMOX1; HNRNPLL; HOXA11-AS; HOXA7; HOXA9; HOXB5; HOXB6; HSD17B6; IFI44; IFIT2; IFIT5; IFITM1; IGSF9; IKBIP; ING1; IRF1; IRX2; IRX3; IRX5; ITPRIP; KB-1460A1.5; KCNS3; KDM6B; KDSR; KIAA0040; KIAA1841; KIF23; KIF25; KIFC1; KITLG; KLF6; KLHL24; KLLN; LFNG; LGI2; LIN9; LINC00662; LINC01011; LINC01089; LINC01315; LMO4; lnc-AL035696.1-5:1; lnc- APOC3-2:1; lnc-C11orf1-1:1; lnc-C17orf62-2:1; lnc-C21orf58-1:1; lnc-C2orf78-1:1; lnc-C6orf192-1:1; lnc-C9orf103-3:1; lnc-C9orf80-1:2; lnc-CCKAR-1:1; lnc-CENPP-1:1; lnc-CLK4-1:1; lnc-COPZ2-1:2; lnc-DNAJC11-1:1; lnc-EIF2D-1:1; lnc-ERP44-3:3; lnc-EYS-2:1; lnc-FAM46D-1:1; lnc-GNS-2:1; lnc-HCRTR1-1:1; lnc-INTS8-1:1; lnc-INTS9-1:3; lnc-IRX3-4:5; lnc-KBTBD6-1:1; lnc-MAPK8IP2-1:12; lnc-MAPK8IP2-1:5; lnc-MCCC2-2:2; lnc-MMRN1-2:6; lnc-MTA2-1:1; lnc-NME4-1:2; lnc-OBFC1-2:2; lnc-OIT3-2:1; lnc-OTOGL-1:1; lnc-PHF3-1:1; lnc-PLEKHA5-1:1; lnc-PRKRIR-1:2; lnc-RALGDS-2:1; lnc-RHOJ-1:1; lnc-RNF125-2:1; lnc-RPLP1-1:14; lnc-RPLP1-1:15; lnc-RPP30-2:1; lnc-RSBN1L-1:1; lnc-SEH1L-1:1; lnc-SEMA6C-1:1; lnc-SFN-1:1; lnc-SOX6-1:1; lnc-SPIRE2-1:1; lnc-TCF19-1:4; lnc-TCL1B-2:1; lnc-TDRD5-1:1; lnc-TTR-1:1; lnc-UQCRFS1-7:1; lnc-ZNF717-1:1; lnc-ZNF74-1:5; lnc-ZSCAN10-3:4; LOC100270804; LOC100288911; LOC100506127; LOC100506302; LOC101929516; LOC388692; LOC399815; LOC400958; LPCAT4; LRIG3; LRP11; LSM3; LTF; MAFB; MAP3K8; MAP7D3; MCM4; MCM9; MEX3B; MNX1; MYO1B; NAA38; NCOA7; NDC1; NEAT1; NEMP1; NET1; NEURL3; NFKBID; NIPAL1; NM_001099435; NR_024011; NR1D1; NR3C1; NRARP; NREP; NRG4; NT5DC3; NUAK2; OAF; ORC1; OSR2; P2RX7; PABPC5; PAG1; PAN3-AS1; PANX1; PDLIM3; PELI1; PER2; PGM2L1; PLEKHO1; PLK2; POGLUT1; POLR3G; PRDM12; PRPF4; PSAT1; PSAT1P1; PSAT1P3; PTCH1; RAB30-AS1; RAP2B; RASIP1; RASSF2; RASSF5; RAVER1; RBM38; RBM43; RDM1; RIPK1; RMI2; RNF125; RNF144B; RNF146; RP11-1007O24.2; RP11-1055B8.4; RP11-150O12.3; RP11-18H7.1; RP11-206L10.9; RP11-227G15.8; RP11-253M7.4; RP11-304L19.12; RP11-363J20.1; RP11-45M22.5; RP11-486G15.2; RP11-57A19.2; RP11-705O1.8; RP13-516M14.1; RTN3; SAV1; SCARNA23; SCGB1D2; SERPINB5; SERPINC1; SESN2; SF3B4; SH3BGRL2; SIX1; SIX4; SLC22A10; SMC6; SNHG21; SNHG4; SNORA61; SOCS1; SOWAHD; SOX8; SPATA41; SPOCK2; SPRED3; ST8SIA4; STIM2; STK38; TAF5L; TAP1; TBX2-AS1; TCONS_l2_00006779; TCONS_l2_00006780; TCONS_l2_00011380; TCONS_l2_00013803; TCONS_l2_00014848; TCONS_l2_00017138; TCONS_l2_00020645; TCONS_l2_00020647; TCONS_l2_00029338; TCONS_l2_00029343; TERF2; TGFBI; TGIF1; THC2601170; THC2674845; TIGD3; TLE1; TNFRSF21; TOR1B; TP53; TPM3; TPTE; TRAF5; TRIB1; TRIM2; TRIM45; TSNAXIP1; TUBBP5; TYMS; TYMSOS; ULBP1; UNC93B1; UQCRB; USP27X; VASH1; VIM-AS1; WEE1; XM_005255122; XR_108380; XR_109753; XR_243166; XR_424187; XR_424475; XR_425268; XR_426337; ZAR1L; ZBTB26; ZFP14; ZNF219; ZNF28; ZNF383; ZNF404; ZNF425; ZNF585A; ZNF713; ZNF79; ZNF845; ZNF850; ZNF878; ZPLD1; ZSCAN12; ZSCAN16-AS1
**510 genes with a significantly downregulated expression by *PVT1* knockdown in androgen treated LNCaP cells (+R1881).**
AC093642.3; ACPP; ADAL; ADCY1; ADGRV1; AF131217.1; AK125979; AL035610.2; ALG14; ANK3; ANKIB1; ANTXR1; AP000473.5; APBA2; ARHGAP29; ARHGAP35;

ARHGAP6; ARHGEF10; ARL8B; ARNT2; ASAP1; ATHL1; ATP2C2; ATXN7L1; AUH; B9D1; BMP6; BTBD9; C11orf80; C5orf28; C8orf37-AS1; CA5BP1; CAB39; CASC4; CCDC88C; CDKAL1; CGNL1; CHMP3; COA1; COMMD1; CRADD; CREBL2; CRYBG3; CSPP1; CUX1; DAPK2; DCAF6; DGCR5; DIAPH2; DIAPH3; DISC1; DLEU1; DOCK1; DOCK4; EFR3A; EIF2AK2; ENST00000612111; EPHA3; EPHB2; ERI3; EXOC4; EXOC7; FAM118A; FAM171A1; FAR2P1; FARP2; FBXL17; FGF13; FGFRL1; FHOD3; FMO4; FOXN3; GALNT10; GALNT16; GFOD1; GLB1L; GMDS; GMDS-AS1; GOLGA8A; GPATCH2; GPC6; GRIK2; GYG2P1; HDAC8; HIVEP3; HOOK1; HS6ST2; ICA1; IMMP2L; INPP4A; INPP5A; JAZF1; KALRN; KCND2; KCNMA1; KDM4B; KDM4C; KIAA0556; KIF13B; LINC00858; LINC00882; LINC01029; LMBR1; lnc-C20orf197-3:13; lnc-EXOC4-1:1; lnc-EXOC4-2:1; lnc-MAGEB10-2:1; lnc-MBL2-2:3; lnc-WRNIP1-2:35; lnc-WRNIP1-2:9; lnc-ZBBX-3:1; LOC100288798; LOC100507291; LOC101927056; LOC101927282; LOC339862; LOC728730; LRRC40; LRRN3; MAP2K4; MAP2K5; MED27; MFF; MIR99AHG; MKL1; MPP6; MSI2; MSRA; MYLK; NAALADL2; NEFH; NLGN1; NLGN4Y; NM_005042; NRXN1; NSMAF; PBX1; PCCA; PDK3; PDPK1; PDSS2; PDZRN3; PIK3CB; PKNOX2; PLCB4; POLA1; POTED; POTEI; PPARA; PPARG; PPM1H; PPP2R2C; PRKCE; PRUNE2; PSMF1; PTPRG; PVT1; RABGAP1; RABGAP1L; RALGPS1; RALYL; RANBP3L; RAPGEF1; ROR1; RP11-122G18.5; RP11-266A24.1; RP11-38M15.11; RPS6KA3; RPS6KA5; RPTOR; RSRC1; RTN4RL1; SBF2; SCFD2; SEMA4F; SEPT11; SESTD1; SETD3; SH3PXD2A; SHANK2; SIL1; SLC24A1; SLC24A3; SMYD3; SNX14; SNX29; SRP14-AS1; SRRM1; SRRM2-AS1; ST3GAL3; STAG2; STK32C; STK33; STYK1; SUSD4; TARSL2; TBC1D22A; TBL1X; TCF4; TCONS_l2_00001492; TCONS_l2_00017189; TCONS_l2_00025015; TENM1; TENM2; TLL1; TMEM222; TMEM242; TMEM266; TMTC2; TNFSF4; TTC28; TTC7B; TYRP1; UBAC2; UBE2E2; USP25; USP31; VAC14; WDR11; WDR7; WNT7B; WWC1; XR_110931; XR_426478; XR_430630; YPEL1; ZBBX; ZBTB20; ZDHHC14; ZFAND3; ZFYVE28; ZNF385B; A_21_P0013943; AC133680.1; ACRV1; ACTN1; ACYP2; ADGRG6; AFF3; AK022088; AK128032; AK130248; AK130387; AK5; AK8; ALCAM; ANKRD20A11P; ANKRD37; AP4S1; APBA1; ARFGEF2; ARHGAP27; ARHGAP28; ARHGAP44; ARHGEF10L; ARHGEF16; ARID1B; ATE1; ATF7; ATG4A; ATP6V0E2-AS1; BC029857; BQ082135; C10orf76; C16orf72; C1orf21; CACNA1H; CAMK2B; CASZ1; CCDC138; CCDC146; CCDC183; CCDC50; CCDC53; CCDC90B; CD99L2; CDC14B; CEP44; CERS6; CHST11; COL27A1; COL4A5; COL4A6; COLEC12; COX7B2; CPAMD8; CPLX3; CPNE7; CPSF1; CREB3L4; CTC-228N24.3; CTD-2384A14.2; CYP4F30P; DCLK2; DENND1A; DENND5B; DEPDC1B; DHRS2; DHX36; DHX37; DIS3L; DISP2; DNAJB12; DNAJC1; DNASE2B; DNTTIP2; DOCK5; DYM; EAF2; EDA; EFNA5; EHMT1; EIF2B3; EIF3H; ENOX1; ENST00000624918; ENTPD5; EVI5; FAM172A; FAM189A1; FAM221A; FAR2P2; FBXO25; FGGY; FMR1NB; FTO; GABPB1; GALNT18; GATS; GNAZ; GOLGA4; GOLGA6A; GOLGA8R; GPR158; GULP1; HHAT; HIF1A; HIPK2; HLCS; HUWE1; IFT140; IGF1R; IL1RAPL1; IRAK1; ITGA9-AS1; KATNAL2; KCNC2; KCNH8; KDM6A; KIAA0355; KIAA1328; KLHL29; KTN1; LA16c-380H5.4; LAMA3; LARP4B; LIN7A; LINC01372; lnc-BAI1-1:1; lnc-FMOD-2:1; lnc-FSIP1-1:1; lnc-MAT2B-3:8; lnc-MIPOL1-2:1; lnc-MIPOL1-4:1; lnc-MYC-2:22; lnc-NLGN1-1:6; lnc-OSBPL9-1:1; lnc-RP3-377D14.1.1-4:1; lnc-SMCR7L-1:1; lnc-SNURF-1:1; lnc-SREK1-2:1; lnc-WRNIP1-2:20; LOC102723927; LOC284930; LOC286437; LOC440910; LPP; LRIG1; LRP8; LRRC20; LTA4H; LUZP2; M6PR; MAML3; MBOAT2; MCTP2; MGAT5; MGC39584; MICAL3; MMP11; MOCOS; MUC4; NCK1-AS1; NEBL; NEK11; NFATC1; NINL; NLGN4X; NME3; NOSTRIN; NPDC1; NPRL3; NRP1; NTNG1; OBSCN; OLA1; OR8A1; OTUD7A; P3H2; PAXBP1-AS1; PEBP4; PI4KA; PI4KAP2; PIGK; PIR; PKN2; PLCB1; POLR1A; POTEB3; POTEM; PRKACB; PRKCH; PRR4; PTPN14;

PTPN21; PTPRM; PTPRN2; PUDP; RALY; RARB; RBKS; REXO2; RFX2; RP11-1038A11.1; RP11-30J20.1; RP11-323I15.2; RP11-476K15.1; RP11-624L4.1; RP11-738O11.9; RP1-65J11.1; RP1-90G24.6; RP4-742C19.12; RXRA; SAMD12; SAP130; SCHLAP1; SENP7; SEPT9; SERGEF; SETBP1; SGMS1; SLC13A3; SLC22A18; SLC22A23; SLC25A21; SLC26A10; SMOC2; SMU1; SPIDR; SPIRE2; SRGAP2; ST3GAL5; ST7; STK39; SYT7; TANGO2; TBC1D1; TBL1Y; TCONS_l2_00000562; TCONS_l2_00001494; TCONS_l2_00017422; TECPR1; TFPI; TG; THBS3; THC2616237; THRB; THSD7A; TMEM229B; TMEM241; TOM1L2; TPT1-AS1; TRAPPC9; TRIM48; TRIM49D1; TRIM53AP; TSHZ3; TTC12; TTC6; U2AF1; UBE4A; UMAD1; VPS37B; VTI1A; XIAP; XM_006720346; XR_241611; XR_424539; YPEL2; ZACN; ZC3H3; ZC4H2; ZDBF2; ZFHX3; ZHX2; ZMIZ1; ZMYM4; ZNF146; ZNF585B; ZNF652

Supplementary Table S4. List of genes with a significant change in the expression level upon *PVT1* knockdown in hormone-deprived cells (-R1881), of which 396 were upregulated and 436 downregulated, compared with their expression in control cells treated with a scrambled GapmeR oligonucleotide (CTRL KD).

**396 genes with a significantly upregulated expression upon *PVT1* knockdown in hormone-deprived LNCaP cells (-R1881)**
A_21_P0014692; A_21_P0014749; A_33_P3300117; A_33_P3367970; A_33_P3484775; AC084809.3; AC087491.2; AK022341; AK055942; AK123993; ALKBH8; AMER1; AOC2; APOE; BC008289; BC110990; BREA2; BZW2; C18orf54; C8G; CCDC177; CDC42EP2; CDK2; CXCR4; DSG1-AS1; EBPL; EID3; ENKD1; ENST00000614063; ERVMER34-1; EXO1; FAHD2A; FJX1; FOXN2; FRMD8; GABPB1-AS1; GAN; GAPLINC; GNRH1; GP1BB; GPR75; HAPLN3; HES7; HIST1H4J; HIST1H4K; HIST2H3A; HSPB2; ICOSLG; IFI6; IGF2BP1; IGFBP5; IHH; IP6K2; ITGA10; KIF18A; KLF4; KLHL11; LINC01167; lnc-ANKH-1:3; lnc-ASH2L-1:1; lnc-CPN2-3:1; lnc-EIF2AK4-4:1; lnc-EIF4EBP1-1:1; lnc-FAM3A-1:1; lnc-GATAD1-2:2; lnc-HLA-DMA-1:1; lnc-NDFIP1-1:1; lnc-PDE2A-1:1; lnc-SNX33-1:1; LOC101927482; LOC101928891; LRRC73; MARVELD1; MDM1; METTL1; MFSD2A; MIR17HG; MSL1; MTMR11; MYBPHL; NAA35; NACC2; NOP56; NR2C2AP; PCDH20; PFKFB2; PIDD1; PNP; POLR1C; PPM1D; PPRC1; PRR22; PSPN; PTGER4; RAB36; RASL11A; RDH10; RDX; RECQL4; RGS16; RNF112; RNU6ATAC; RP11-1275H24.1; RP11-159D12.2; RP11-359M6.3; RP11-655M14.13; RP4-564F22.5; RPL32; SCARF2; SCARNA11; SCARNA14; SCARNA18; SCARNA3; SGK223; SGOL1; SKIL; SLC17A4; SLC1A3; SNHG15; SNHG17; SNORA13; SNORA14B; SNORA2A; SNORA30; SNORA36B; SNORA38; SNORA40; SNORA41; SNORA44; SNORA46; SNORA50C; SNORA57; SNORA5A; SNORA5B; SNORA63; SNORA65; SNORA68; SNORA74B; SNORA79; SNORA80A; SNORA80B; SNORA84; SNORD104; SNORD62A; SNORD64; SNORD76; SNORD99; SPEF1; STX2; TCL1B; TCONS_l2_00000969; TCONS_l2_00006103; TCONS_l2_00019714; TCP10L; TFF1; TGM1; THC2756939; TPM2; VTRNA1-2; VTRNA1-3; VWCE; ZMYND10; ZNF169; ZNF577; A_33_P3223860; A_33_P3301876; A_33_P3345031; AC024560.2; ACRC; ACTA1; ADAM20P1; ADRB1; AEN; AK025118; AK096098; AK123926; AK127152; ALB; ANXA1; ANXA2R; AREG; ARL4A; ARRDC2; ASH1L-AS1; AURKAPS1; BBC3; BRD8; BRDT; BRI3; BTN3A1; BTN3A2; C1orf56; C2orf72; C2orf81; C4orf46; CARD9; CCNE2; CD3EAP; CD55; CDH26; CEACAM1; CEP135; CHAC1; CHRNA10; CLDN11; COX19; CTD-2292M16.8; CTXN1; CYP27A1; DCUN1D5; DDIAS; DHX58; DIEXF; DLX2; DNAAF3; DNHD1; DOK3; DTL; DTWD1; DUSP18; DYRK3; E2F7; ECM1; EFCAB3; EGR1; ENKUR; ERAP2; ESPL1; ESPNL; FAM155B; FAM187A; FAM209A; FAM46C; FAM71A; FAM81A; FAM84A; FAM87A; FAS; FAXDC2; FDXR; FGD3; FOXD4L5; FOXN4; FOXQ1; GADD45A; GATA6; GLS; GPRIN1; GPX8; GRM2; GSG2; GUCY1B2; HCLS1; HRK; IDI2-AS1; IER5; IFFO1; IFIT1; IL20RB; IQCD; IRS1; KCNIP2-AS1; KCNJ14; KIAA0408; KIAA1462; KIF2C; KLHL31; LAMB2P1; LIF; LINC00324; LINC00476; LINC01311; LINC01410; LMO2; lnc-AK5-1:1; lnc-AP000769.1-1:1; lnc-CERK-1:1; lnc-DCTD-1:1; lnc-DKK4-1:1; lnc-FAM72C-2:1; lnc-FAM98A-1:1; lnc-IDE-1:1; lnc-MATN2-2:1; lnc-MMRN1-2:1; lnc-MMRN1-2:2; lnc-MMRN1-2:5; lnc-NDST2-3:1; lnc-NT5DC3-1:1; lnc-OBFC2A-1:1; lnc-PITPNC1-1:1; lnc-PPM1D-1:1; lnc-RPL7L1-1:1; lnc-SEPT7L-1:1; lnc-SERPINC1-1:24; lnc-SLC39A8-1:1; lnc-SRBD1-1:1; lnc-TAF15-1:1; lnc-WNT1-2:1; lnc-WRAP73-1:1; LOC100506804; MCM10; MDM2; MESDC1; MIA; MORF4L2-AS1; MPZL2; MRM1; MS4A8; MTFR2; MTHFD2; MYB; NAB2; NABP1; NOV; NR2F1; ORC6; PAX1; PAX2; PDE4A; PDE4D; PDE4DIP; PGF; PHLDA1; PHLDA3;

PIM2; PLAC8L1; PLK3; PMAIP1; PMP22; PPP1R27; PRKAG2-AS1; PTGES2-AS1; PTGFR; RASL11B; RBM26-AS1; REM2; RHBDF2; RHEBL1; RIN1; RNF19B; RP11-318C24.2; RP11-344N10.5; RP11-395G23.3; RP11-621L6.3; RPA4; RPRML; RRN3P2; SBSN; SCARNA8; SKA1; SLC18B1; SLC25A19; SLC25A36; SLC29A1; SLC7A2; SNHG1; SNHG12; SNORA26; SNORA3B; SNORA8; SNORD103A; SNORD21; SNORD43; SNORD47; SNORD84; SNORD86; SOGA3; SPG7; SPRY1; STC2; TBX2; TCONS_l2_00009373; TCONS_l2_00020648; TFAP2A-AS1; THC2667604; THC2724751; TICRR; TIGAR; TLR3; TMED6; TMEM243; TNFSF10; TNFSF9; TOR4A; TSPAN12; TTR; UBAP2; UHRF1; WDR66; XR_241982; ZC3H12C; ZFP36L2; ZGLP1; ZNF124; ZNF215; ZNF436-AS1
**436 genes with a significantly downregulated expression by *PVT1* knockdown in hormone-deprived LNCaP cells (-R1881).**
A_21_P0014213; A_33_P3222600; AC010967.2; AC098973.2; ACAT1; ACER3; ACTN3; ADAMTS17; ADD1; AFF1; AGAP1; AIDA; AK123308; AK130873; AL117431; AMN1; ANKRD20A12P; ANKRD20A2; ANKRD44; ANXA6; APMAP; ARL15; ARMC9; ASAP2; ASTN2; ATP9B; BCAS1; BCAS3; BLMH; C1orf95; CA12; CAMKMT; CFAP44; CHCHD3; CNBD1; COLQ; CTD-2001C12.1; CTD-2151A2.1; CTPS2; CUEDC1; DDO; DENND1B; DEPTOR; DIO3; DIS3L2; DPP4; DSCR3; EEF1A1; EEFSEC; EML5; ERC1; ERCC6L2; ERGIC1; ERMAP; FAM3B; FARP1; FBLN7; FBXL7; FER; FOXO4; FRMD5; FRMPD2; FZD6; GALNT11; GAS6; GBA3; GOLGA6L4; GOLGA6L9; GOLGA8J; GPA33; GRIK1; GRK5; GUCD1; HIRA; HIST2H2BE; INADL; ITPK1; ITPR1; KCNAB2; KCNK13; KCNU1; KDM5B; KIAA1033; KIAA1217; KLHL13; LACE1; LARGE; LHPP; LINC00565; LINC01006; LINC01354; LINGO3; lnc-ADAM30-1:1; lnc-ARID2-3:1; lnc-C11orf39-3:1; lnc-C17orf46-1:1; lnc-HIATL1-1:1; lnc-PEX2-3:1; lnc-PLCD1-1:6; lnc-TRAP1-1:1; lnc-TRH-2:1; lnc-WDR7-6:1; LOC100130587; LOC101929633; LOC401052; LOC553103; LRP1; LRRC42; LYPD6B; MAGI1; MAPK10; MATN2; MBD5; MECOM; MEGF8; MFN2; MFSD8; NACAD; NAP1L4; NCAPG2; NDRG3; NEU4; NIPAL3; NIPSNAP3B; NRG3; NXN; OPRK1; OSBP2; OTUD5; PALLD; PANK3; PITPNM3; PKIB; PLA2G4F; PLCG2; PLEKHB2; PLXDC2; PPFIA1; PPIP5K1; PTK2; PTPN13; PTPRR; RAP2C-AS1; ROR2; RP11-166P13.3; RP11-305L7.1; RP11-319F12.2; RP11-379B8.1; RP11-452F19.3; RP11-456O19.2; RP11-548L20.1; RP11-666A20.4; RP11-723O4.6; RPS6KA2; RRBP1; RTN1; RYR2; SDC3; SEC23A; SECISBP2; SHISA3; SHROOM2; SLC16A7; SLC38A6; SLCO5A1; SMARCB1; SMPDL3A; SNAP47; SNRK; SORBS2; SPG11; SSH1; STAG3L4; STIM1; STX18-AS1; STXBP4; STYXL1; SYT17; TCONS_l2_00012688; TCONS_l2_00016154; TCONS_l2_00018853; TCONS_l2_00018976; TGM3; TJP2; TMEM210; TMEM245; TNIK; TPK1; TRAM2; TRMT2B; TSPAN9; TXNRD2; TYW1; VPS13A; WNK2; XR_242359; XR_242407; XR_246983; XR_425926; XR_428452; XR_431240; ZAK; ZBED3-AS1; ZMAT1; AC093642.3; ACPP; ADAL; ADCY1; ADGRV1; AF131217.1; AK125979; AL035610.2; ALG14; ANK3; ANKIB1; ANTXR1; AP000473.5; APBA2; ARHGAP29; ARHGAP35; ARHGAP6; ARHGEF10; ARL8B; ARNT2; ASAP1; ATHL1; ATP2C2; ATXN7L1; AUH; B9D1; BMP6; BTBD9; C11orf80; C5orf28; C8orf37-AS1; CA5BP1; CAB39; CASC4; CCDC88C; CDKAL1; CGNL1; CHMP3; COA1; COMMD1; CRADD; CREBL2; CRYBG3; CSPP1; CUX1; DAPK2; DCAF6; DGCR5; DIAPH2; DIAPH3; DISC1; DLEU1; DOCK1; DOCK4; EFR3A; EIF2AK2; ENST00000612111; EPHA3; EPHB2; ERI3; EXOC4; EXOC7; FAM118A; FAM171A1; FAR2P1; FARP2; FBXL17; FGF13; FGFRL1; FHOD3; FMO4; FOXN3; GALNT10; GALNT16; GFOD1; GLB1L; GMDS; GMDS-AS1;

GOLGA8A; GPATCH2; GPC6; GRIK2; GYG2P1; HDAC8; HIVEP3; HOOK1; HS6ST2; ICA1; IMMP2L; INPP4A; INPP5A; JAZF1; KALRN; KCND2; KCNMA1; KDM4B; KDM4C; KIAA0556; KIF13B; LINC00858; LINC00882; LINC01029; LMBR1; lnc-C20orf197-3:13; lnc-EXOC4-1:1; lnc-EXOC4-2:1; lnc-MAGEB10-2:1; lnc-MBL2-2:3; lnc-WRNIP1-2:35; lnc-WRNIP1-2:9; lnc-ZBBX-3:1; LOC100288798; LOC100507291; LOC101927056; LOC101927282; LOC339862; LOC728730; LRRC40; LRRN3; MAP2K4; MAP2K5; MED27; MFF; MIR99AHG; MKL1; MPP6; MSI2; MSRA; MYLK; NAALADL2; NEFH; NLGN1; NLGN4Y; NM_005042; NRXN1; NSMAF; PBX1; PCCA; PDK3; PDPK1; PDSS2; PDZRN3; PIK3CB; PKNOX2; PLCB4; POLA1; POTED; POTEI; PPARA; PPARG; PPM1H; PPP2R2C; PRKCE; PRUNE2; PSMF1; PTPRG; PVT1; RABGAP1; RABGAP1L; RALGPS1; RALYL; RANBP3L; RAPGEF1; ROR1; RP11-122G18.5; RP11-266A24.1; RP11-38M15.11; RPS6KA3; RPS6KA5; RPTOR; RSRC1; RTN4RL1; SBF2; SCFD2; SEMA4F; SEPT11; SESTD1; SETD3; SH3PXD2A; SHANK2; SIL1; SLC24A1; SLC24A3; SMYD3; SNX14; SNX29; SRP14-AS1; SRRM1; SRRM2-AS1; ST3GAL3; STAG2; STK32C; STK33; STYK1; SUSD4; TARSL2; TBC1D22A; TBL1X; TCF4; TCONS_l2_00001492; TCONS_l2_00017189; TCONS_l2_00025015; TENM1; TENM2; TLL1; TMEM222; TMEM242; TMEM266; TMTC2; TNFSF4; TTC28; TTC7B; TYRP1; UBAC2; UBE2E2; USP25; USP31; VAC14; WDR11; WDR7; WNT7B; WWC1; XR_110931; XR_426478; XR_430630; YPEL1; ZBBX; ZBTB20; ZDHHC14; ZFAND3; ZFYVE28; ZNF385B

Supplementary Table S5. List of 160 genes in LNCaP cells with expression significantly downregulated by androgen stimulation in the presence of endogenous levels of *PVT1* and with their expression upregulated upon *PVT1* knockdown in androgen treated cells, plus the list of TCGA-PRAD gene IDs for 121 of these genes.

**160 genes with expression significantly downregulated by androgen, which became upregulated upon *PVT1* knockdown**
C4orf46; CCNE2; CDH26; CHAC1; FAS; FGD3; FOXN4; FOXQ1; GATA6; GPX8; GUCY1B2; HRK; IER5; KIAA0408; KIAA1462; LIF; LINC00476; lnc-FAM72C-2:1; lnc-SRBD1-1:1; MYB; NABP1; NOV; PAX1; PDE4D; PDE4DIP; PMP22; PRKAG2-AS1; RASL11B; SLC18B1; SLC7A2; SOGA3; TCONS_l2_00020648; TFAP2A-AS1; TSPAN12; A_33_P3346526; ACKR3; ACTA2; ADM; AGBL5-AS1; AJUBA; AK124361; AMIGO3; ANKRD16; ANP32E; ARHGAP32; ARL4D; ASB13; ASNSP1; BIRC3; BMF; C10orf10; CBLN2; CBR3; CCNO; CD83; CERK; CLCN5; CNKSR3; COLCA1; DAND5; DDX11L16; DNAH11; DRAIC; DSC2; ENC1; ENST00000623723; EXPH5; F2R; FAM198B; FAM212B; FAM217B; FAM229B; FGD6; FOXD3; FOXO4; FOXO6; FSCN1; GIMAP2; GJB2; GPAT3; HAGLROS; HEG1; HOXA11-AS; HOXA7; HOXA9; HOXB5; HOXB6; IFIT2; IFIT5; IFITM1; IRF1; IRX3; IRX5; KCNS3; KIF25; KITLG; KLHL24; KLLN; LFNG; lnc-CCKAR-1:1; lnc-EYS-2:1; lnc-INTS8-1:1; lnc-INTS9-1:3; lnc-IRX3-4:5; lnc-OIT3-2:1; lnc-PHF3-1:1; lnc-RPLP1-1:14; lnc-RPLP1-1:15; lnc-ZNF74-1:5; LOC100288911; LOC400958; LPCAT4; LRIG3; LTF; MAP3K8; MEX3B; MNX1; NCOA7; NET1; NEURL3; NIPAL1; NR_024011; NR3C1; NREP; NUAK2; OSR2; P2RX7; PABPC5; PAG1; PAN3-AS1; PDLIM3; PGM2L1; PRDM12; PSAT1; PSAT1P1; PSAT1P3; RAP2B; RASSF2; RASSF5; RDM1; RNF125; RNF144B; RP11-253M7.4; RP11-45M22.5; RP11-57A19.2; SCGB1D2; SERPINB5; SH3BGRL2; SLC22A10; SMC6; ST8SIA4; TCONS_l2_00017138; TCONS_l2_00029343; THC2601170; TLE1; TNFRSF21; TRIB1; TRIM2; TRIM45; XR_424475
**121 genes (out of the 160 genes above) that were present in the TCGA prostate adenocarcinoma 2016 (PRAD) dataset**
1: C4orf46; 2: CCNE2; 3: CDH26; 4: CHAC1; 5: FAS; 6: FGD3; 7: FOXN4; 8: FOXQ1; 9: GATA6; 10: GPX8; 11: GUCY1B2; 12: HRK; 13: IER5; 14: KIAA0408; 15: KIAA1462; 16: LIF; 17: C9orf130; 18: MYB; 19: OBFC2A; 20: NOV; 21: PAX1; 22: PDE4D; 23: PDE4DIP; 24: PMP22; 25: RASL11B; 26: C6orf192; 27: SLC7A2; 28: C6orf174; 29: TSPAN12; 30: ACTA2; 31: ADM; 32: JUB; 33: AMIGO3; 34: ANKRD16; 35: ANP32E; 36: ARHGAP32; 37: ARL4D; 38: ASB13; 39: BIRC3; 40: BMF; 41: C10orf10; 42: CBLN2; 43: CBR3; 44: CCNO; 45: CD83; 46: CERK; 47: CLCN5; 48: CNKSR3; 49: DAND5; 50: DNAH11; 51: DSC2; 52: ENC1; 53: EXPH5; 54: F2R; 55: FAM198B; 56: C1orf183; 57: C20orf177; 58: C6orf225; 59: FGD6; 60: FOXD3; 61: FOXO4; 62: FSCN1; 63: GIMAP2; 64: GJB2; 65: AGPAT9; 66: HEG1; 67: HOXA11AS; 68: HOXA7; 69: HOXA9; 70: HOXB5; 71: HOXB6; 72: IFIT2; 73: IFIT5; 74: IFITM1; 75: IRF1; 76: IRX3; 77: IRX5; 78: KCNS3; 79: KIF25; 80: KITLG; 81: KLHL24; 82: KILLIN; 83: LFNG; 84: LPCAT4; 85: LRIG3; 86: LTF; 87: MAP3K8; 88: MEX3B; 89: MNX1; 90: NCOA7; 91: NET1; 92: NEURL3; 93: NIPAL1; 94: NR3C1; 95: C5orf13; 96: NUAK2; 97: OSR2; 98: P2RX7; 99: PABPC5; 100: PAG1; 101: PDLIM3; 102: PGM2L1; 103: PRDM12; 104: PSAT1; 105: RAP2B; 106: RASSF2; 107: RASSF5; 108: RDM1; 109: RNF125; 110: RNF144B; 111: SCGB1D2; 112: SERPINB5; 113: SH3BGRL2; 114: SLC22A10; 115: SMC6; 116: ST8SIA4; 117: TLE1; 118: TNFRSF21; 119: TRIB1; 120: TRIM2; 121: TRIM45
